# Synthesis of *N*-Difluoromethyl Benzothiazole (or Benzoxazole) Selenones as Novel Inhibitors Against Phytopathogenic Fungi

**DOI:** 10.3390/molecules31020314

**Published:** 2026-01-16

**Authors:** Zihao Huang, Zhen Liu, Baixin Zhang, Jing Jiao, Ri-Yuan Tang

**Affiliations:** 1 Key Laboratory of Advanced Materials for Facility Agriculture, Ministry of Agriculture, College of Materials and Chemical Engineering, South China Agricultural University, Guangzhou 510642, Chinalzhen202302@163.com (Z.L.);; 2 School of Chemistry and Civil Engineering, Shaoguan University, Shaoguan 512005, China

**Keywords:** thiazole, oxazole, selenium, antifungal agent, phytopathogenic fungi

## Abstract

Azole selenoureas exhibit diverse biological functions. However, the synthesis and biological activity of benzothiazole and benzoxazole selenones remained unexplored. Herein, we report the base-catalyzed synthesis of *N*-difluoromethyl benzothiazole (or benzoxazole) selenone derivatives, which demonstrated significant antifungal efficacy against *Rhizoctonia solani*, *Phytophthora infestans*, *Botrytis cinerea*, and *Fusarium oxysporum*. Compound **3b** exhibited exceptional antifungal activity against *R. solani*, with an EC_50_ of 2.10 mg/L. Moreover, it substantially inhibited sclerotia germination (81.5% at 9 mg/L) and formation (79.3% at 9 mg/L), surpassing octhilinone. The protective effect on detached rice leaves and rice seedlings was found to be 43.4% and 85.2% at 100 mg/L, respectively, and 64.4% and 89.4% at 200 mg/L. These findings suggest that benzothiazole and benzoxazole selenones represent promising lead compounds for sustainable plant disease management.

## 1. Introduction

**Figure 1:**
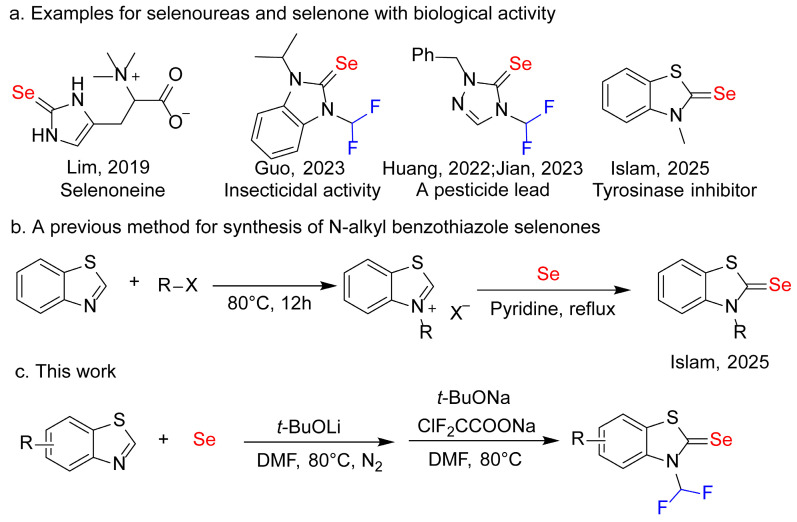
Bioactive molecules and synthetic methods of benzothiazole selenones [4,5,6,16,17].

Phytopathogenic fungi represent a significant challenge to global agricultural productivity, inducing substantial yield reductions and economic losses [1]. Single-pathway fungicidal interventions have frequently precipitated resistant strain development, underscoring the critical necessity for innovative antifungal agents with novel chemical architectures and multi-mechanistic action modalities [2]. Organic selenium compounds have garnered substantial research interest due to their diverse biological efficacies [3]. For example, selenourea derivatives demonstrate pronounced antifungal, insecticidal, and plant growth modulation properties [4,5,6,7,8]. Selenium atoms participate in redox cycles, generating reactive oxygen species (ROS) that induce oxidative stress, DNA damage, and apoptosis in target organisms [9,10,11]. As an essential trace element for numerous biological systems, selenium’s strategic incorporation into agrochemicals can provide dual benefits of crop protection and nutritional enhancement [12,13,14,15]. Imidazole selenoureas are a promising class of organoselenides found in the natural product Seleoneine (Figure 1a) [16], which is derived from deep-sea fish and exhibits excellent antioxidant ability. These compounds exhibit enhanced bioavailability and multifunctional bioactivity due to the combination of the selenourea pharmacophoric moiety and a heterocyclic framework [4,5,6]. Previous investigations have elucidated that these compounds can inhibit fungal proliferation, compromise insect midgut structural integrity, and serve as selenium nutritional sources for botanical systems [4,5,6]. Our research group has developed *N*-difluoromethyl-substituted azole selenoureas as artificial organoselenide defense (AOSeD) agents [4,5,6]. These fluoroazole selenoureas (FASU) and triazole selenoureas demonstrate broad-spectrum efficacy against agricultural pests *Plutella xylostella* [4], phytopathogenic fungi (*Rhizoctonia solani*, *Colletotrichum higginsianum*), and weeds [5]. Moreover, these compounds function as nutritional fortifiers, enhancing soluble proteins, sugars, flavonoids, phenolic acids, glucosinolates, and essential mineral elements in microgreens [6].

Despite recent advancements, synthetic methodologies for *N*-fluoroalkyl azole selenoureas remain constrained [18,19,20]. The prevailing approach predominantly involves the condensation of azoles with bromodifluoroacetate and elemental selenium under sulfite-mediated conditions [18]. However, this strategy has been primarily limited to imidazole and triazole scaffolds, leaving *N*-difluoromethyl benzothiazole and benzoxazole selenones substantially unexplored. Although there is a synthetic method for *N*-alkyl benzothiazole selenones involving the reaction of *N*-alkyl benzothiazole salts with selenium (Figure 1b) [17], it is not suitable for the synthesis of *N*-difluoromethyl benzothiazole selenones. The paucity of efficient synthetic routes to such heterocyclic selenones impedes comprehensive evaluation of their potential biological applications. The expansion of the selenone library to incorporate benzothiazole and benzoxazole derivatives represents a significant research trajectory. These heterocyclic scaffolds are pivotal structural motifs in medicinal and agricultural chemistry, characterized by enhanced molecular bioavailability, versatile non-covalent interaction capabilities, and optimized pharmacokinetic characteristics [21,22,23,24,25,26]. The incorporation of selenone unit into these molecular scaffolds may generate compounds exhibiting enhanced target affinity, distinctive mechanistic profiles, and potentially superior antifungal efficacy. The synergistic integration of a selenium-based redox-active center within a fused bicyclic system could potentially provide multi-site inhibitory mechanisms, thereby mitigating potential resistance development.

This study aims to develop synthetic routes for *N*-difluoromethyl benzothiazole and benzoxazole selenones (Figure 1c), and assess their antifungal efficacy against predominant phytopathogenic fungi. The research will investigate in vitro and in vivo inhibitory efficacy of these compounds against *R. solani*, *P. infestans*, *B. cinerea*, and *F. oxysporum*. By analyzing structure-activity relationships, this work seeks to identify novel lead compounds for sustainable plant disease management, advancing the development of multifunctional agrochemicals that enhance crop protection and plant health.

## 2. Results

### 2.1. Synthesis of N-Difluoromethyl Benzothiazole (or Benzoxazole) Selenones

**Figure 2:**
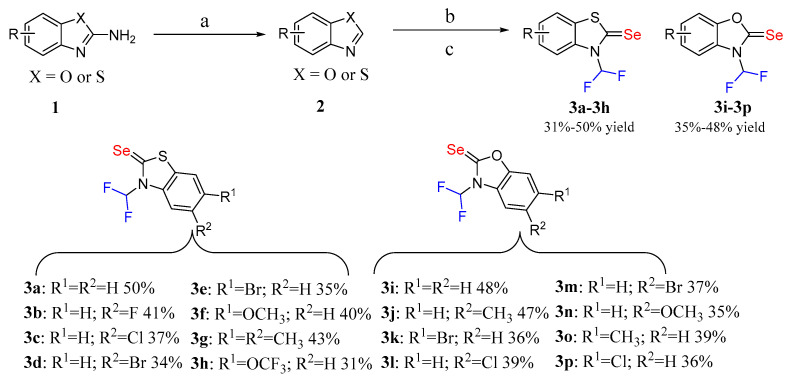
Synthesis of *N*-difluoromethyl benzothiazole (or benzoxazole) selenones: (a) *t*-BuONO, THF, 35 °C, 6 h; (b) *t*-BuOLi, Se, DMF, N_2_, 80 °C, 12 h; (c) Sodium chlorodifluoroacetate, *t*-BuONa, DMF, 80 °C, 12 h.

As shown in Figure 2, the synthetic pathway for *N*-difluoromethyl benzothiazole (or benzoxazole) selenones **3a**–**3p** involves a two-step procedure. Building upon previous research methodologies [27,28], benzothiazoles (or benzoxazoles) **2** were synthesized via the deamination of 2-amino benzothiazole (or benzoxazole) using *t*-BuONO in THF at 35 °C for 6 h. Subsequently, the benzothiazoles or benzoxazoles **2** were reacted with Se and *t*-BuOLi in anhydrous DMF under N_2_ atmosphere at 80 °C for 12 h, yielding benzothiazole (or benzoxazole) selenones. The in situ generated selenones were then reacted with sodium chlorodifluoroacetate in the presence of *t*-BuONa in DMF at 80 °C for 12 h, affording *N*-difluoromethyl benzothiazole (or benzoxazole) selenones **3a**–**3p** in moderate yields. Notably, benzothiazoles and benzoxazoles exhibit lower reactivity compared to imidazoles and triazoles, rendering them incompatible with the previous one-pot synthetic method [18,19]. The structures of compounds **3a**–**3p** were confirmed using nuclear magnetic resonance (NMR) and high-resolution mass spectrometry (HRMS) technologies. For the ^1^H NMR signal peaks, F_2_HC- exhibits coupling triplet signal peaks at approximately δ 8.30 for compounds **3a**–**3h** and δ 7.85 for the remaining compounds. The coupling constant of F–H is approximately 58.0 Hz. The ^13^C NMR of F_2_HC- also exhibits coupling triplet peaks at approximately δ 111.5, with a coupling constant of F–C ranging from 250.0 to 260.0 Hz. The selenone carbon adjacent to F_2_HC- exhibits coupling triplet peaks at 180.0–190.0 Hz, with a coupling constant of approximately 3.0 Hz (see Supporting Information).

### 2.2. Antifungal Activity

Four phytopathogenic fungi, including *R. solani*, *P. infestans*, *B. cinerea*, and *F. oxysporum*, were evaluated for antifungal activity of compounds **3a**–**3p** at 25 mg/L (Table 1). Octhilinone and azoxystrobin were used as the positive controls. Compounds **3a**–**3p** demonstrated significant inhibitory effects against the tested fungi. Compounds **3a**, **3b**, **3f**, **3k**, **3l**, **3n**, **3i**, **3j** and **3p** completely inhibited *R. solani*, which was comparable to octhilinone. Against *P. infestans*, compounds **3a** and **3b** achieved 100% inhibition, whereas compounds **3f**, **3h**, **3k**, **3l**, **3n**, **3o**, and **3p** showed >50% inhibitory activity. For *B. cinerea*, compounds **3a**, **3b**, **3f**, **3k**, and **3l** displayed >50% inhibition. *F. oxysporum* inhibition > 50% was observed with compounds **3a**, **3b**, **3f**, **3h**–**3l**, and **3n**–**3p**. Notably, compounds **3a** and **3b** demonstrated the most consistent and robust antifungal efficacy across all four fungal species. However, azoxystrobin, the positive control, showed poor antifungal activity towards the four phytopathogenic fungi.

The effective concentration 50% (EC_50_) values of these compounds against four phytopathogenic fungi were evaluated (Table 2). Most compounds demonstrated significant antifungal activity, particularly against *R. solani*. Compounds **3a** and **3b** exhibited exceptional antifungal efficacy across all four fungi, with EC_50_ values below 10 mg/L. Their EC_50_ for *R. solani* were 2.50 mg/L and 2.10 mg/L, respectively, marginally higher than octhilinone (EC_50_ = 1.18 mg/L). Compound **3b**, featuring a fluoro substituent, displayed superior activity against *R. solani* compared to chloro or bromo-substituted analogs (**3c** and **3d**). Methoxy and trifluoromethoxy substituents on the benzene ring enhanced antifungal activity (compound **3f**, EC_50_ 2.59 mg/L; compound **3h**, EC_50_ 2.56 mg/L). Among benzoxazole selenones, unsubstituted compound **3i** demonstrated notable activity against *R. solani* (EC_50_ 5.24 mg/L). Halogenated compounds **3k** and **3p** exhibited improved antifungal properties (EC_50_ 3.71 mg/L and 2.24 mg/L, respectively). Compounds **3j** and **3n**, bearing methyl or methoxy groups, also demonstrated favorable antifungal activities (EC_50_ 3.07 mg/L and 3.91 mg/L, respectively). The substitution site also affects the antifungal activity (EC_50_: 3.71 mg/L for compound **3k** vs. 11.9 mg/L for compound **3m**; 6.35 mg/L for compound **3l** vs. 2.24 mg/L for compound **3p**; 3.07 mg/L for compound **3j** vs. 14.5 mg/L for compound **3o**).

### 2.3. Determination of Active Functional Groups

**Figure 3:**
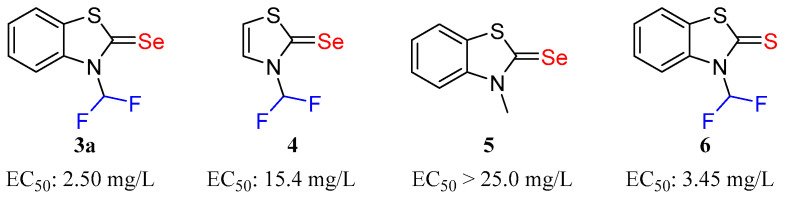
Molecular structure of compounds **3a**, **4**, **5** and **6** and EC_50_ against *R. solani*.

To elucidate the effects of the conjugated plane, difluoromethyl, and selenium on the antifungal activity of compound **3a**, the biological assays of compounds **4**, **5**, and **6** were conducted and compared with that of compound **3a** (Figure 3). Detailed synthetic methods for compounds **4**, **5**, and **6** can be found in the section of Synthetic Procedures. Compound **3a**, featuring a benzene ring, exhibited superior efficacy compared to compound **4**, suggesting the benzene ring with π electrons can enhance antifungal activity. Substitution of the difluoromethyl group with a methyl group in compound **5** significantly diminished antifungal potency, indicating the difluoromethyl moiety is critical for high activity. Benzothiazole thiourea **6** demonstrated notable inhibitory activity against *R. solani*, with an EC_50_ of 3.45 mg/L, lower than compound **3a** (2.50 mg/L). The selenium atom appears to contribute to the compound’s antifungal efficacy [29,30]. It can be concluded that the benzene ring, the selenium atom, and the difluoromethyl group all contribute to the high antifungal activity.

### 2.4. Effect of Compound ***3b*** on the Formation and Germination of R. solani Sclerotium

**Figure 4:**
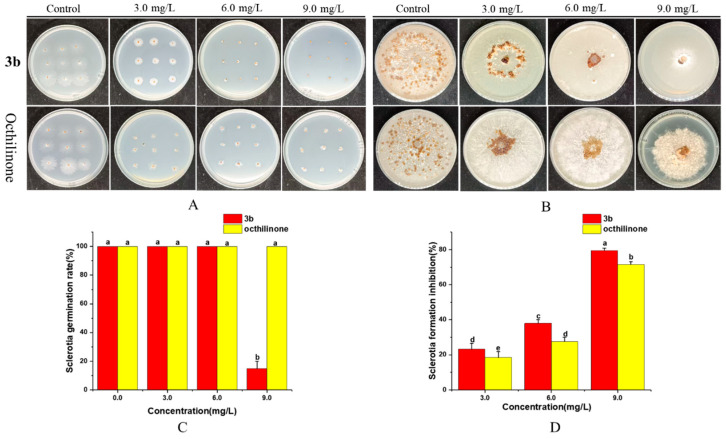
Effects of compound **3b** on the sclerotia germination (**A**,**C**) and formation (**B**,**D**) of *R. solani.* The ANOVA was significant for a, b, c, d and e. Means were separated using the least significant difference test (LSD, *p* < 0.05).

*R. solani* is a destructive phytopathogen infecting over 27 plant families, causing significant agricultural losses [31]. As a critical rice sheath blight pathogen, it can induce yield reductions ranging from 2.5% to 50%, potentially exceeding 50% in susceptible varieties [32,33]. Inhibiting the formation and germination of sclerotium can effectively prevent and control the harm of *R. solani*. Therefore, the inhibitory effect of compound **3b** on *R. solani* sclerotium formation and germination was evaluated (Figure 4). Compared to the control, both compound **3b** and octhilinone demonstrated inhibitory effects, though neither completely prevented sclerotia germination at 3.0 and 6.0 mg/L. At 9.0 mg/L, compound **3b** significantly inhibited sclerotia germination with an 81.5% inhibitory rate, whereas octhilinone remained ineffective. Compound **3b** exhibited superior inhibitory effects on sclerotia formation at 3.0, 6.0, and 9.0 mg/L, with inhibitory rates of 23.2%, 38.1%, and 79.3%, respectively. This antifungal lead compound significantly suppresses both sclerotial germination and formation of *R. solani*, outperforming the positive control with octhilinone. It offers a promising alternative for the sustainable management of rice sheath blight [34].

### 2.5. In Vivo Antifungal Activity Against R. solani of Compound ***3b***

**Figure 5:**
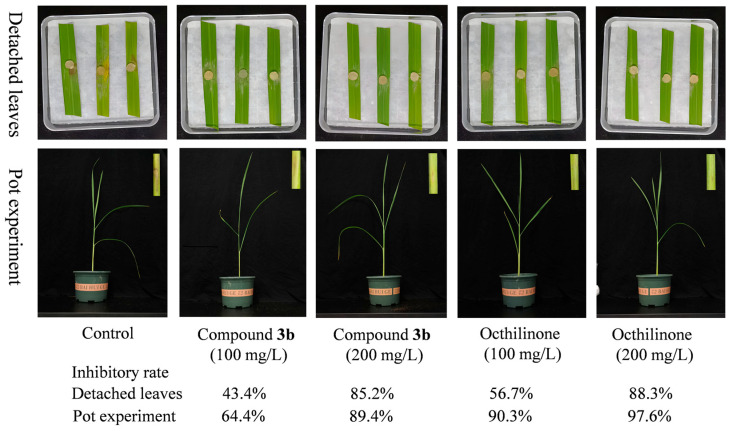
In vivo antifungal activity of compound **3b** against *R. solani*.

In protective assays on excised rice leaves, compound **3b** exhibited antifungal activity against *R. solani*. Treated leaves showed significantly reduced disease symptoms compared to the control. The protective effect of **3b** was concentration-dependent, with suppression rates reaching 43.4% at 100 mg/L and 85.2% at 200 mg/L, comparable to those of octhilinone (56.7% and 88.3%, respectively). In pot experiments evaluating inhibition on rice leaf sheaths, with octhilinone as a positive control (Figure 5), untreated plants developed extensive brown lesions. In contrast, **3b** treatment markedly suppressed lesion expansion, showing protective efficacy of 64.4% at 100 mg/L and 89.4% at 200 mg/L, though slightly lower than that of octhilinone.

Building on its previously reported ability to inhibit sclerotia, **3b** demonstrates notable control efficacy against rice sheath blight in both detached leaf and whole-plant assays. These results underscore its potential for protecting rice during susceptible growth stages and support its value as a practical candidate for integrated disease management.

## 3. Discussion

### 3.1. Synthesis of N-Difluoromethyl Benzothiazole and Benzoxazole Selenones

This study successfully established a novel synthetic route for a series of *N*-difluoromethyl benzothiazole and benzoxazole selenones (**3a**–**3p**). The developed two-step synthetic protocol effectively overcomes the previously reported limitation of applying one-pot methods to less nucleophilic benzothiazole and benzoxazole cores. The successful synthesis of these compounds confirms the adaptability of the base-catalyzed selenation/fluoroalkylation strategy to a wider range of azole systems, thereby significantly enlarging the library of accessible “artificial organoselenide defense” (AOSeD) agents [4,5,6,7,8]. This methodological advance is crucial for future structure-activity relationship (SAR) explorations in this chemical class.

### 3.2. Analysis of Biological Active Groups

The determination of active functional groups provided critical mechanistic insights. As shown in Figure 3, the significant reduction in activity when the difluoromethyl group was replaced with a methyl group (compound **5**) highlights the importance of this motif, which is likely due to its strong electron-withdrawing nature and its ability to participate in specific dipole–dipole interactions or act as a weak hydrogen bond donor. The retained, albeit reduced, activity of the thione analogue **6** compared to selenone **3a** strongly supports the critical role hypothesised for the selenium atom. This is consistent with previous studies suggesting that the selenium centre in selenoureas participates in redox cycling by generating reactive oxygen species (ROS), which induce oxidative stress in fungal cells [7]. The superior activity of the selenone suggests that the enhanced redox activity and possibly different interaction geometry of selenium compared to sulfur contribute to greater antifungal efficacy. The observation that the benzene-fused system (**3a**) is more active than the non-fused analogue (compound **4**) highlights the importance of molecular planarity and potential for π-π stacking interactions with aromatic residues in target biomolecules, thereby enhancing binding affinity.

The electronic properties and lipophilicity of the substituents significantly affect antifungal activity. Compounds **3b** and **3h** contain a fluoro or a trifluoromethyl group, which can improve lipophilicity and lead to increased permeability. These compounds can also act as hydrogen bonding acceptors, enhancing binding affinity with biomolecules. Halo and halo-containing groups may form halogen bonds with the target biomolecules. This is why they are much more effective than the nonpolar methyl group, which does not easily interact with biological molecules. Interestingly, the methoxy group, an electron-donating group, is much more effective than the methyl group. It may increase the electron density of the conjugated plane, thereby improving π-π stacking interactions with target biomolecules.

### 3.3. Antifungal Efficacy

The most compelling results pertain to the biological impact on *R. solani*, a notoriously difficult-to-control soil-borne pathogen. Compound **3b** demonstrated an exceptional ability to suppress not just mycelial growth but also the critical survival and reproductive structures—sclerotia. Its superior inhibition of both sclerotial germination and formation at 9 mg/L, outperforming octhilinone, suggests a mode of action that disrupts fundamental developmental or metabolic pathways essential for the pathogen survival and dormancy. This multi-pronged inhibitory effect is highly desirable for resistance management, as it likely imposes a higher fitness cost on the fungus to develop evasion strategies.

The in vivo protective efficacy of **3b** on both detached leaves and whole rice seedlings further validates its potential as a practical agrochemical lead. The concentration-dependent reduction in disease severity, achieving protection rates > 85% at 200 mg/L, translates the promising in vitro activity into a relevant pathological context. Although it is slightly less effective than octhilinone in some assays, its novel structure and unique combination of antifungal and anti-sclerotial activity give it a profile that is distinct profile from that of existing fungicides. The effective concentration of compound **3b** and Octhilinone in the in vivo assay is much higher than that in the in vitro assay. This is because the in vitro assay was conducted in a culture plate that could maintain a certain level of humidity, and the antifungal agent solution was distributed evenly across the plate. This enabled molecules to penetrate the mycelium tissues more easily. In contrast, when the antifungal agent solution was sprayed onto rice leaves, the solvent easily evaporated, leaving the antifungal agent in powder form on the leaf surface. In this form, the antifungal agent could not easily penetrate the mycelium tissues. This is why a high concentration was required for the in vivo assay.

In the broadest sense, these findings reinforce the strategic importance of incorporating selenium into the design of agrochemicals. As hypothesized, the combination of the selenium-based redox mechanism and the optimized pharmacokinetic profile provided by the benzothiazole/oxazole scaffold appears to be successful. This class of compounds meets the growing demand for multifunctional crop protection agents that also promote plant health, given the beneficial role of selenium as a nutrient [6].

## 4. Materials and Methods

### 4.1. Chemicals and Instruments

Unless otherwise specified, chemicals (≥97% purity) were acquired from commercial sources without further purification. Fungi: *Fusarium oxysporum*; *Rhizoctonia solani*; *Phytophthora infestans*; *Botrytis cinerea* procured from the College of Plant Protection at South China Agricultural University was cultured on potato dextrose agar and preserved at 4 °C.

All ^1^H NMR and ^13^C NMR spectra were measured with a Bruker Avance-III 500 instrument (Billerica, MA, USA, 500 MHz for ^1^H, 125 MHz for ^13^C NMR spectroscopy) using tetramethylsilane (TMS) as a reference. High-resolution mass spectra (HRMS) were obtained via electrospray ionization (ESI) using a Waters G2-XS Q-TOF LC/MS mass spectrometer (Milford, MA, USA).

### 4.2. Synthetic Procedures

#### 4.2.1. Synthetic Procedure for Intermediates **2** [27,28]

At 0 °C, a reaction mixture comprising 2-aminobenzothiazole/benzoxazole (1 mmol), *t*-BuONO (2 mmol), and THF (2 mL) was added sequentially to a 15 mL reaction vessel with a Teflon-lined screw cap which was purchased from Chongqing Xinwei Glass Co., Ltd. (Chongqing, China). The mixture was stirred at 35 °C for 12 h, with reaction progress monitored by thin-layer chromatography. The reaction mixture was washed with ethyl acetate and filtered under vacuum. The filtrate was adsorbed onto 200–300 mesh silica gel by solvent evaporation under reduced pressure. The crude product was purified via column chromatography on silica gel using a hexane/ethyl acetate (volume ratio: 20/1) eluent gradient. The purified compound was subsequently dried under vacuum.

#### 4.2.2. Synthesis of *N*-Difluoromethyl Thiazole (or Benzoxazole) Selenones

Under a nitrogen atmosphere, a sealed 15 mL tube equipped with a magnetic stirrer was charged with benzoxazole/thiazole (0.40 mmol), *t*-BuOLi (0.80 mmol, 64.0 mg, 2 equiv), and selenium (0.80 mmol, 63.2 mg, 2.0 equiv). Anhydrous DMF (2.0 mL) was added. The tube was sealed and heated at 80 °C for 12 h. Subsequently, sodium chlorodifluoroacetate (0.80 mmol, 122.8 mg, 2 equiv), *t*-BuONa (0.80 mmol, 76.9 mg, 2 equiv), and anhydrous DMF (1.0 mL) were introduced. The reaction mixture was maintained at 80 °C for 12 h, with reaction progress monitored by thin-layer chromatography. Upon completion, the crude reaction mixture was diluted with ethyl acetate, filtered through a diatomaceous earth pad, and extracted using a separatory funnel. The organic layer was washed with saturated saline solution, dehydrated with diatomaceous earth and anhydrous sodium sulfate, and concentrated under reduced pressure. The resulting residue was purified via flash column chromatography using petroleum ether and ethyl acetate as eluents.

#### 4.2.3. Synthesis of Compounds **4**, **5**, and **6**

Compound **4** was prepared using the previously described method. The detailed synthetic procedure can be found in reference [20]. The detailed synthetic procedure for compound **5** can be found in reference [17]. The synthesis of compound **6** is the same as that of *N*-difluoromethyl benzothiazole (or benzoxazole) selenones.

### 4.3. Antifungal Activity Assay In Vitro [7,35]

The antifungal activity was evaluated employing the mycelial linear growth rate methodology. Fungal strains maintained at 4 °C were cultured on potato dextrose agar (PDA). Heterocycle-fused FASU was dissolved in dimethyl sulfoxide (DMSO) and incorporated into PDA to generate media with varying concentrations in 90-mm Petri dishes. The final DMSO concentration was 0.5%, which did not significantly affect fungal growth. Accordingly, 0.5% DMSO in PDA served as the negative control, while PDA supplemented with octhilinone and 0.5% DMSO (*v*/*v*) was used as the positive control. A 0.6-cm mycelial disc of the test fungus was centrally inoculated on the experimental and control media.

Samples were measured in triplicate, and inhibition zone diameters (mm) were measured using the cross-bracketing method. The inhibition rate was calculated using the formula:

(C − T)/(C − 0.6) × 100(1)

where C and T represent mean colony diameters of control and treatment groups, respectively. Compounds exhibiting inhibition rates > 70% during preliminary screening at 25 mg/L were selected for half maximal effective concentration (EC_50_) determination, with each assay performed in triplicate.

### 4.4. Inhibition Assay on Sclerotia Formation and Germination [7,35]

#### 4.4.1. Inhibition Assay on Sclerotia Formation

Compound **3b** and octhilinone were dissolved in dimethyl sulfoxide (DMSO) and incorporated into potato dextrose agar (PDA) at concentrations of 3.0, 6.0 and 9.0 mg/L. *R. solani* mycelial disks (0.6 cm diameter) were inoculated into PDA supplemented with varying compound **3b** concentrations and incubated at 28 °C in darkness for 12 days. Formed sclerotia were collected, desiccated at 60 °C for 36 h, and quantified. The sclerotia formation inhibition rate was calculated using the formula: (M0 − Mt)/M0 × 100, where M0 and Mt represent mean sclerotia weights in control and treatment groups, respectively. Octhilinone served as the positive control. Experiments were conducted in triplicate.

#### 4.4.2. Inhibition Assay on Sclerotia Germination

After cultivation, *R. solani* sclerotia were individually placed on culture media supplemented with diverse concentrations of compound **3b**. The treatments were incubated at 28 °C for 24 h, and the germination inhibition rate was subsequently determined. Octhilinone served as the positive control, with each concentration evaluated in triplicate.

### 4.5. In Vivo Inhibition Assay of Compound ***3b*** [7,35]

A controlled environment pot experiment was conducted to evaluate the in vivo biocontrol efficacy of compound **3b** against *R. solani* in rice plants at the tillering stage. The compound was dissolved in dimethyl sulfoxide and diluted with 0.1% Tween 80 aqueous solution, which exhibited no phytotoxicity. The protective effects of compound **3b** were assessed on in vitro rice leaves and potted rice plants, with each experimental treatment replicated thrice.

In the protection assay, rice leaf sheaths were pre-treated with compound **3b** at 50 and 100 mg/L concentrations, utilizing octhilinone as a positive control and Tween 80 aqueous solution as a negative control. Mycelial plugs were subsequently inoculated into the treated leaf sheaths and incubated at 25 °C for 50 h. In the curative assay, mycelial plugs were initially introduced into rice leaf sheaths. After 48 h of incubation, the leaf sheaths were treated with compound **3b** and further incubated at 25 °C for 48 h. Each experiment was performed in triplicate.

Moreover, the protective characteristics of isolated rice leaves corroborate the preceding findings. The experiment was replicated, and the results were analyzed using Image Pro Plus 6.0. The protective and healing efficacies of compound **3b** were quantified through the following formula:

[(S^0^ − S^1^)/S^0^] × 100 (2)

where S^0^ represents the lesion area in the control group and S^1^ represents the lesion area in the treatment group.

### 4.6. Statistical Analyses

Antifungal activity assays were performed in triplicate, with results expressed as mean ± standard deviation. Statistical analyses were conducted using SPSS 25.0 and Origin 2018.

## 5. Conclusions

A synthetic method for *N*-difluoromethyl benzothiazole (or benzoxazole) selenones was developed through the reaction of benzothiazole (or benzoxazole) with Se and *t*-BuLi at elevated temperatures. This study has successfully bridged a synthetic gap and unveiled a novel class of *N*-difluoromethyl benzothiazole/benzoxazole selenones with significant antifungal and anti-sclerotial properties. Compound **3b** was identified as a highly effective lead candidate due to its potent, broad-spectrum antifungal activity against key phytopathogens, particularly *R. solani* (EC_50_ = 2.10 mg/L). Its ability to suppress the germination and formation of sclerotia, a critical pathogenic factor, is superior, and it demonstrates significant protective efficacy in both detached leaf and whole-plant assays. These results highlight its potential for use in crop protection, offering a new chemical tool born from the rational integration of selenium chemistry and privileged heterocyclic scaffolds. These findings conclusively demonstrate that this novel class of selenone compounds is a promising basis for developing new, sustainable antifungal agents.

## Figures and Tables

**Table 1:** Antifungal activity of compounds against four phytopathogenic fungi.

Compound (25 mg/L)	Average Inhibition Rat ± SD (%) (*n* = 3)			
	*R. solani*	*P. infestans*	*B. cinerea*	*F. oxysporum*
**3a**	100 ± 0.0	100 ± 0.0	97.6 ± 0.0	100 ± 0.0
**3b**	100 ± 0.0	100 ± 0.0	79.5 ± 0.3	87.8 ± 0.1
**3c**	58.0 ± 0.7	15.1 ± 1.0	28.5 ± 0.4	21.5 ± 0.5
**3d**	15.9 ± 0.2	0.0 ± 0.0	21.9 ± 0.1	4.6 ± 0.4
**3e**	63.6 ± 0.5	36.4 ± 0.2	35.1 ± 0.4	37.1 ± 0.0
**3f**	100 ± 0.0	73.3 ± 0.6	57.5 ± 0.5	63.4 ± 0.4
**3g**	42.3 ± 0.2	5.3 ± 0.1	17.6 ± 0.2	8.9 ± 0.2
**3h**	94.4 ± 0.3	64.3 ± 0.3	35.1 ± 0.9	64.5 ± 0.6
**3i**	100 ± 0.0	45.0 ± 0.7	24.2 ± 0.0	56.6 ± 0.1
**3j**	100 ± 0.0	49.2 ± 0.5	15.3 ± 0.0	54.6 ± 0.3
**3k**	100 ± 0.0	58.5 ± 0.4	64.1 ± 0.5	60.9 ± 0.2
**3l**	100 ± 0.0	52.4 ± 0.2	53.3 ± 0.0	56.9 ± 0.9
**3m**	79.0 ± 0.3	38.1 ± 0.6	8.8 ± 0.2	40.0 ± 0.4
**3n**	100 ± 0.0	57.7 ± 0.2	19.0 ± 0.4	70.8 ± 0.0
**3o**	65.5 ± 0.1	60.3 ± 0.3	25.3 ± 0.0	56.1 ± 0.0
**3p**	100 ± 0.0	65.9 ± 0.1	49.4 ± 0.0	62.9 ± 0.8
Octhilinone	100 ± 0.0	83.8 ± 0.4	94.2 ± 0.0	78.2 ± 0.2
Azoxystrobin	71.0 ± 0.2	25.7 ± 0.7	65.3 ± 0.4	62.0 ± 0.8

**Table 2:** EC_50_ for four phytopathogenic fungi.

Compound	EC_50_ (mg/L)			
	*R. solani*	*P. infestans*	*B. cinerea*	*F. oxysporum*
**3a**	2.50	4.69	7.68	5.25
**3b**	2.10	6.31	7.49	5.73
**3c**	20.00	>25.0	>25.0	>25.0
**3d**	>25.0	>25.0	>25.0	>25.0
**3e**	15.7	>25.0	>25.0	>25.0
**3f**	2.59	9.56	21.5	15.9
**3g**	>25.0	>25.0	>25.0	>25.0
**3h**	2.56	15.20	>25.0	16.2
**3i**	5.24	>25.0	>25.0	20.0
**3j**	3.07	>25.0	>25.0	22.1
**3k**	3.71	17.6	14.6	18.5
**3l**	6.35	19.7	24.5	21.5
**3m**	11.9	>25.0	>25.0	>25.0
**3n**	3.91	21.1	>25.0	11.7
**3o**	14.5	16.9	>25.0	21.01
**3p**	2.24	13.3	>25.0	17.1
Octhilinone	1.18	2.77	1.09	3.13
Azoxystrobin	3.51	>25.0	12.8	13.5

## Data Availability

The raw data supporting the conclusions of this article will be made available by the authors on request.

## Supplementary Materials

The following supporting information can be downloaded at: https://www.mdpi.com/article/10.3390/molecules31020314/s1. Structural characterization data and NMR spectra are available in the Supporting Information.
