# Supplementary material for: Synthesis of N-Difluoromethyl Benzothiazole (or Benzoxazole) Selenones as Novel Inhibitors Against Phytopathogenic Fungi
Source: Molecules. 2026 Jan 16;31(2):314. doi: 10.3390/molecules31020314 (PMC12843817; doi:10.3390/molecules31020314)
Supplement: Supplementary file 1 [file molecules-31-00314-s001.zip › molecules-4093386-supplementary.pdf]

Supporting Information

Synthesis of *N*-Difluoromethyl Thiazole (or Benzoxazole) Selenones as Novel Inhibitors Against Phytopathogenic Fungi

Zihao Huang,[1,†] Zhen Liu,[1,†] Baixin Zhang,[1] Jing Jiao[2,*] and Ri-Yuan Tang[1,*]

[1]Key Laboratory of Advanced Materials for Facility Agriculture, Ministry of Agriculture,College of Materials and Chemical Engineering, South China Agricultural University, Guangzhou 510642, China

[2] School of Chemistry and Civil Engineering, Shaoguan University, Shaoguan 512005, China

[*]Corresponding authors, E-mail address: rytang@scau.edu.cn, jiaojing@sgu.edu.cn

contents

1. Structural characterization data of compounds 2

2. NMR spectra of compounds 8

1. Structural characterization data for compounds 3a-3p, 4, 5 and 6

3-(difluoromethyl)benzo[d]thiazole-2(3H)-selenone (3a)

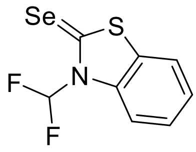

Yellow solid. M.P.: 77-80 °C. Yield: 50%. ^{1}H NMR (500 MHz, $CDCl_3$) δ 8.33 (t, J = 57.7 Hz, 1H), 7.78 (d, J = 8.1 Hz, 1H), 7.54-7.39 (m, 3H). ^{13}C NMR (125 MHz, $CDCl_3$) δ 188.2 (t, J = 3.2 Hz, 1C), 138.6, 128.9, 127.6, 126.1, 121.1, 113.8 (t, J = 4.5 Hz, 1C), 111.71 (t, J = 256.1 Hz, 1C). HRMS (ESI): m/z: calcd for $C_8H_5F_2NSSe$, $[M+H]^+$: 265.9349, found: 265.9335.

3-(difluoromethyl)-5-fluorobenzo[d]thiazole-2(3H)-selenone (3b)

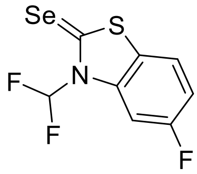

Yellow solid. M.P.: 106-108 °C. Yield: 41%. ^{1}H NMR (500 MHz, $CDCl_3$) δ 8.29 (t, J = 57.7 Hz, 1H), 7.49 (m, 2H), 7.18 (m, 1H). ^{13}C NMR (125 MHz, $CDCl_3$) δ 189.2 (t, J = 2.7 Hz, 1C), 162.2 (d, J = 248.2, 1C), 139.3 (d, J = 12.6 Hz, 1C), 124.1 (d, J = 2.7 Hz, 1C), 121.9 (d, J = 9.6 Hz, 1C), 114.1 (d, J = 25.2 Hz, 1C), 111.6 (t, J = 257.0 Hz, 1C), 101.9 (dt, J = 29.0, 4.8 Hz, 1C). HRMS (ESI): m/z: calcd for $C_8H_4F_3NSSe$, $[M+H]^+$: 283.9255, found: 283.9244.

4-chloro-3-(difluoromethyl)benzo[d]thiazole-2(3H)-selenone (3c)

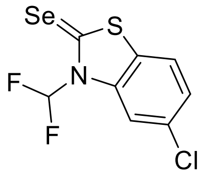

Yellow solid. M.P.: 96-98 °C. Yield: 37%. ^{1}H NMR (500 MHz, $CDCl_3$) δ 8.28 (t, J = 57.6 Hz, 1H), 7.77 (s, 1H), 7.44 (d, J = 8.1 Hz, 1H), 7.39 (dd, J = 8.1, 1.2 Hz, 1H). ^{13}CNMR (125 MHz, $CDCl_3$) δ188.6 (t, J = 3.0 Hz, 1C), 139.3, 134.2, 127.2, 126.5, 121.6, 114.0 (t, J = 4.9 Hz, 1C), 111.5 (t, J = 256.0 Hz, 1C). HRMS (ESI): m/z: calcd for $C_8H_4ClF_2NSSe$, $[M+H]^+$: 299.8960, found: 299.8946.

5-bromo-3-(difluoromethyl)benzo[d]thiazole-2(3H)-selenone (3d)

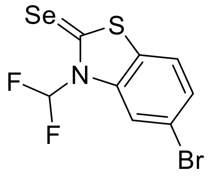

Yellow solid. M.P.: 165-169 °C. Yield: 34%. ^{1}H NMR (500 MHz, $CDCl_3$) δ 8.26 (t, *J* = 57.7 Hz, 1H), 7.92 (d, *J* = 1.8 Hz, 1H), 7.54 (dd, *J* = 7.9, 1.9 Hz, 1H), 7.92 (d, *J* = 7.9 Hz, 1H). ^{13}C NMR (125 MHz, $CDCl_3$) δ 188.4 (t, *J* = 3.0 Hz, 1C), 139.5, 129.3, 127.8, 121.9, 121.7, 116.8 (t, *J* = 4.7 Hz, 1C), 111.6 (t, *J* = 256.4 Hz, 1C). HRMS (ESI): m/z: calcd for $C_8H_4BrF_2NSSe$, $[M+H]^+$: 343.8454, found: 343.8442.

6-bromo-3-(difluoromethyl)benzo[d]thiazole-2(3H)-selenone (3e)

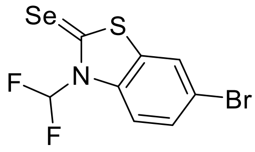

Yellow solid. M.P.: 113-115 °C. Yield: 35%. ^{1}H NMR (500 MHz, $CDCl_3$) δ 8.27 (t, *J* = 57.7 Hz, 1H), 7.67 (s, 1H), 7.62 (d, *J* = 8.8 Hz, 1H), 7.55 (d, *J* = 8.7 Hz, 1H). ^{13}C NMR (125 MHz, $CDCl_3$) δ 187.9 (t, *J* = 2.8 Hz), 137.7, 130.8, 130.7, 123.7, 119.9, 114.6 (t, *J* = 4.7 Hz, 1C), 111.6 (t, *J* = 256.6 Hz, 1C). HRMS (ESI): m/z: calcd for $C_8H_4BrF_2NSSe$, $[M+H]^+$: 343.8454, found: 343.8468.

3-(difluoromethyl)-6-methoxybenzo[d]thiazole-2(3H)-selenone (3f)

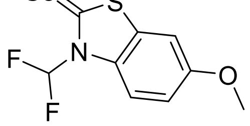

Yellow solid. M.P.: 109-111 °C. Yield: 40%. ^{1}H NMR (500 MHz, $CDCl_3$) δ 8.27 (t, *J* = 57.9 Hz, 1H), 7.66 (d, *J* = 9.8 Hz, 1H), 7.01 (m, 2H), 3.85 (s, 3H). ^{13}C NMR (125 MHz, $CDCl_3$) δ 186.9 (t, *J* = 3.2 Hz, 1C), 158.3, 132.6, 130.4, 115.1, 114.4 (t, *J* = 4.6 Hz, 1C), 111.6 (t, *J* = 255.8 Hz,1C), 104.9, 55.9. HRMS (ESI): m/z: calcd for $C_9H_7F_2NOSSe$, $[M+H]^+$: 295.9455, found: 295.9546.

4-(difluoromethyl)-5,6-dimethylbenzo[d]thiazole-2(3H)-selenone (3g)

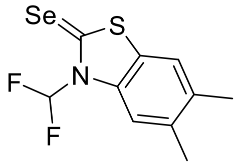

Yellow solid. M.P.: 146-149 °C. Yield: 43%. ^{1}H NMR (500 MHz, $CDCl_3$) δ 8.30 (t, *J* = 57.9 Hz, 1H), 7.55 (s, 1H), 7.27 (s, 1H), 2.37 (s, 3H), 2.32 (s, 3H). ^{13}C NMR (125 MHz, $CDCl_3$) δ 187.6 (t, *J* = 3.78 Hz, 1C), 137.1, 137.0, 135.5, 126.1, 121.2, 114.4 (t, *J* = 4.41 Hz, 1C), 111.7 (t, *J* = 255.8 Hz, 1C), 20.3, 19.8. HRMS (ESI): m/z: calcd for $C_{10}H_9F_2NSSe$, $[M+H]^+$: 293.9662, found: 293.9653.

3-(difluoromethyl)-6-(trifluoromethoxy)benzo[d]thiazole-2(3H)-selenone (3h)

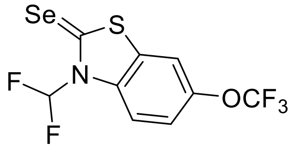

Yellow solid. M.P.: 90-93 °C. Yield: 32%. ^{1}H NMR (500 MHz, $CDCl_3$) δ 8.28 (t, *J* = 57.7 Hz, 1H), 7.77 (d, *J* = 8.9 Hz, 1H), 7.41 (s, 1H), 7.32 (d, *J* = 8.8 Hz , 1H). ^{13}C NMR (125 MHz, $CDCl_3$) δ 188.4 (t, *J* = 3.0 Hz), 147.3, 137.1, 130.4, 120.3 (q, *J* = 259.6 Hz, 1C), 120.8, 114.4 (t, *J* = 4.7 Hz, 1C), 113.9, 111.6 (t, *J* = 257.0 Hz, 1C). HRMS (ESI): m/z: calcd for $C_9H_4F_5NOSSe$, $[M+H]^+$: 349.9172, found: 349.9165.

4-(difluoromethyl)benzo[d]oxazole-2(3H)-selenone (3i)

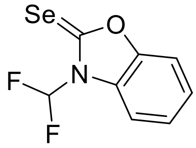

White solid. M.P.: 90-93 °C. Yield: 48%. 1H NMR (500 MHz, $CDCl_3$) δ 7.87 (t, *J* = 58.2 Hz, 1H), 7.54–7.37 (m, 4H). ^{13}C NMR (125 MHz, $CDCl_3$) δ 180.4 (t, *J* = 3.7 Hz, 1C), 148.8, 127.5, 126.0, 125.9, 111.9 (t, *J* = 253.8 Hz, 1C), 111.5 (t, *J* = 2.6 Hz, 1C), 110.94. HRMS (ESI): m/z: calcd for $C_8H_9F_2NOSe$, $[M+H]^+$: 249.9578, found: 249.9580.

3-(difluoromethyl)-5-methylbenzo[d]oxazole-2(3H)-selenone (3j)

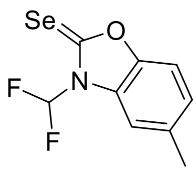

White solid. M.P.: 135-137 °C. Yield: 47%. ^{1}H NMR (500 MHz, $CDCl_3$) δ 7.86 (t, *J* = 58.2 Hz, 1H), 7.32 (d, *J* = 8.7 Hz, 2H), 7.17 (d, *J* = 9.1 Hz, 1H), 2.47 (s, 3H). ^{13}C NMR (125 MHz, $CDCl_3$) δ 180.4 (t, *J* = 3.7 Hz, 1C), 147.1, 136.5, 127.5, 126.7, 111.9 (t, *J* = 253.3 Hz, 1C), 111.7 (t, *J* = 2.5 Hz, 1C), 110.4, 21.4. HRMS (ESI): m/z: calcd for $C_9H_7F_2NOSe$, $[M+H]^+$: 263.9734, found: 263.9722.

5-bromo-3-(difluoromethyl)benzo[d]oxazole-2(3H)-selenone (3k)

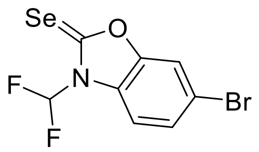

White solid. M.P.: 130-133 °C. Yield: 36%. ^{1}H NMR (500 MHz, $CDCl_3$) δ 7.82 (t, *J* = 58.1 Hz, 1H), 7.62 (d, *J* = 1.6 Hz, 1H), 7.51 (dd, *J* = 8.5, 1.7 Hz, 1H), 7.39 (d, *J* = 8.5 Hz, 1H). ^{13}C NMR (125 MHz, $CDCl_3$) δ 180.4 (t, *J* = 3.5 Hz), 149.2, 129.2, 126.9, 119.2, 114.4, 112.2 (t, *J* = 2.7 Hz, 1C), 111.8 (t, *J* = 254.5 Hz, 1C).HRMS (ESI): m/z: calcd for $C_8H_4BrF_2NOSe$, $[M+H]^+$: 327.8683, found: 327.8668.

6-chloro-3-(difluoromethyl)benzo[d]oxazole-2(3H)-selenone (3l)

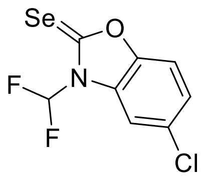

White solid. M.P.: 133-136 °C. Yield: 39%. ^{1}H NMR (500 MHz, $CDCl_3$) δ 7.82 (t, *J* = 58.1 Hz, 1H), 7.53 (s, 1H), 7.41 – 7.34 (m, 2H). ^{13}C NMR (125 MHz, $CDCl_3$) δ 180.7 (t, *J* = 3.4 Hz, 1C), 147.4, 132.1, 128.4, 126.2, 111.8 (t, *J* = 2.5 Hz, 1C), 111.7 (t, *J* = 254.5 Hz, 1C), 111.6. HRMS (ESI): m/z: calcd for $C_8H_4ClF_2NOSe$, $[M+H]^+$: 283.9188, found: 283.9174.

4-bromo-3-(difluoromethyl)benzo[d]oxazole-2(3H)-selenone (3m)

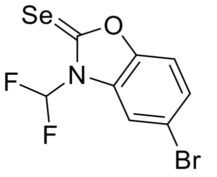

White solid. M.P.: 170-173 °C. Yield: 37%.^{1}H NMR (500 MHz, $CDCl_3$) δ 7.82 (t, *J* = 58.1 Hz, 1H), 7.67 (s, 1H), 7.51 (dd, *J* = 8.7, 1.7 Hz, 1H), 7.33 (d, *J* = 8.7 Hz, 1H). ^{13}C NMR (125 MHz, $CDCl_3$) δ 180.6 (t, *J* = 3.5 Hz), 147.9, 129.0, 128.8, 119.1, 114.5 (t, *J* = 2.9 Hz, 1C), 112.0, 111.7 (t, *J* = 255.2 Hz, 1C). HRMS (ESI): m/z: calcd for $C_8H_4BrF_2NOSe$, $[M+H]^+$: 327.8683, found: 327.8670.

3-(difluoromethyl)-5-methoxybenzo[d]oxazole-2(3H)-selenone (3n)

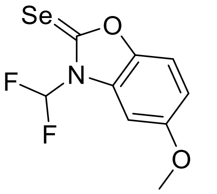

White solid. M.P.: 112-115 °C. Yield: 35%.^{1}H NMR (500 MHz, $CDCl_3$) δ 8.27 (t, *J* = 57.9 Hz, 1H), 7.66 (d, *J* = 9.8 Hz, 1H), 7.01 (d, *J* = 1.6 Hz, 1H), 6.90 (dd, *J* = 9.8, 1.6 Hz, 1H), 3.85 (s, 3H). ^{13}C NMR (125 MHz, $CDCl_3$) δ 186.9 (t, *J* = 3.2 Hz, 1C), 158.3, 132.6, 130.4, 115.1, 114.4 (t, *J* = 4.6 Hz, 1C), 111.6 (t, *J* = 255.8 Hz, 1C), 104.9, 56.2. HRMS (ESI): m/z: calcd for $C_9H_7F_2NO_2Se$, $[M+H]^+$: 279.9683, found: 279.9670.

4-(difluoromethyl)-6-methylbenzo[d]oxazole-2(3H)-selenone (3o)

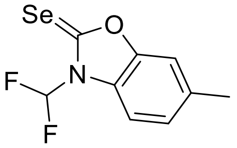

White solid. M.P.: 132-135 °C. Yield: 39%. ^{1}H NMR (500 MHz, $CDCl_3$) δ 7.84 (t, *J* = 58.3 Hz, 1H), 7.39 (d, *J* = 8.2 Hz, 1H), 7.26 (s, 1H), 7.18 (d, *J* = 8.2 Hz, 1H), 2.46 (s, 3H). ^{13}C NMR (125 MHz, $CDCl_3$) δ 180.2 (t, *J* = 3.73 Hz, 1C), 149.1, 136.9, 126.7, 125.3, 111.9 (t, *J* = 253.9 Hz, 1C), 111.2, 110.9 (t, *J* = 2.48 Hz, 1C), 21.5. HRMS (ESI): m/z: calcd for $C_9H_7F_2NOSe$, $[M+H]^+$: 263.9743, found: 263.9730.

5-chloro-3-(difluoromethyl)benzo[d]oxazole-2(3H)-selenone (3p)

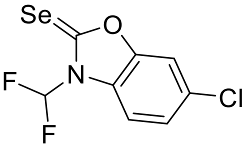

White solid. M.P.: 114-116 °C. Yield: 36%. ^{1}H NMR (500 MHz, $CDCl_3$) δ 7.82 (t, *J* = 58.1 Hz, 1H), 7.48 (d, *J* = 1.8 Hz, 1H), 7.46 (d, *J* = 8.6 Hz, 1H), 7.37 (dd, *J* = 8.6, 1.75 Hz, 1H). ^{13}C NMR (125 MHz, $CDCl_3$) δ 180.5 (t, *J* = 3.6 Hz, 1C), 149.1, 132.2, 126.4, 126.3 (2C), 111.8 (t, *J* = 2.5 Hz), 111.7 (t, *J* = 253.9 Hz, 1C). HRMS (ESI): m/z: calcd for $C_8H_4ClF_2NOSe$, $[M+H]^+$: 283.9188, found: 283.9175.

4-(difluoromethyl)thiazole-2(3H)-selenone (4)

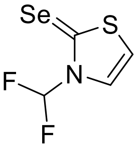

Brown liquid. Yield: 67%. ^{1}H NMR (500 MHz, $CDCl_3$) *δ* 7.89 (t, *J* = 59.3 Hz, 1H), 7.43 (d, *J* = 4.6 Hz, 1H), 6.92 (d, *J* = 4.6 Hz, 1H); ^{13}C NMR (125 MHz, $CDCl_3$) *δ* 184.2 (t, *J* = 2.09 Hz, 1C), 127.2, 116.5, 109.8 (t, *J* = 257.2 Hz, 1C). HRMS (ESI): m/z: calcd for $C_4H_3F_2NSSe$, $[M+H]^+$: 215.9193, found: 215.9198.

5-(difluoromethyl)benzo[d]thiazole-2(3H)-thione (6)

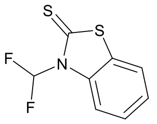

Yellow solid. M.P.: 77-80 °C. Yield: 63%. ^{1}H NMR (500 MHz, $CDCl_3$) δ 8.19 (t, *J* = 57.5 Hz, 1H), 7.70 (d, *J* = 6.6 Hz, 1H), 7.51 – 7.43 (m, 2H), 7.38 (d, *J* = 7.5Hz, 1H). ^{13}C NMR (125 MHz, $CDCl_3$) δ 191.42 (t, *J* = 2.9 Hz), 137.17, 127.42, 126.00, 125.77, 121.26, 113.71 (t, *J* = 4.5 Hz, 1C), 109.90 (t, *J* = 253.6 Hz, 1C). HRMS (ESI): m/z: calcd for $C_8H_5F_2NS_2$, $[M+H]^+$: 217.9905, found: 217.9914.

2. NMR spectra of compounds

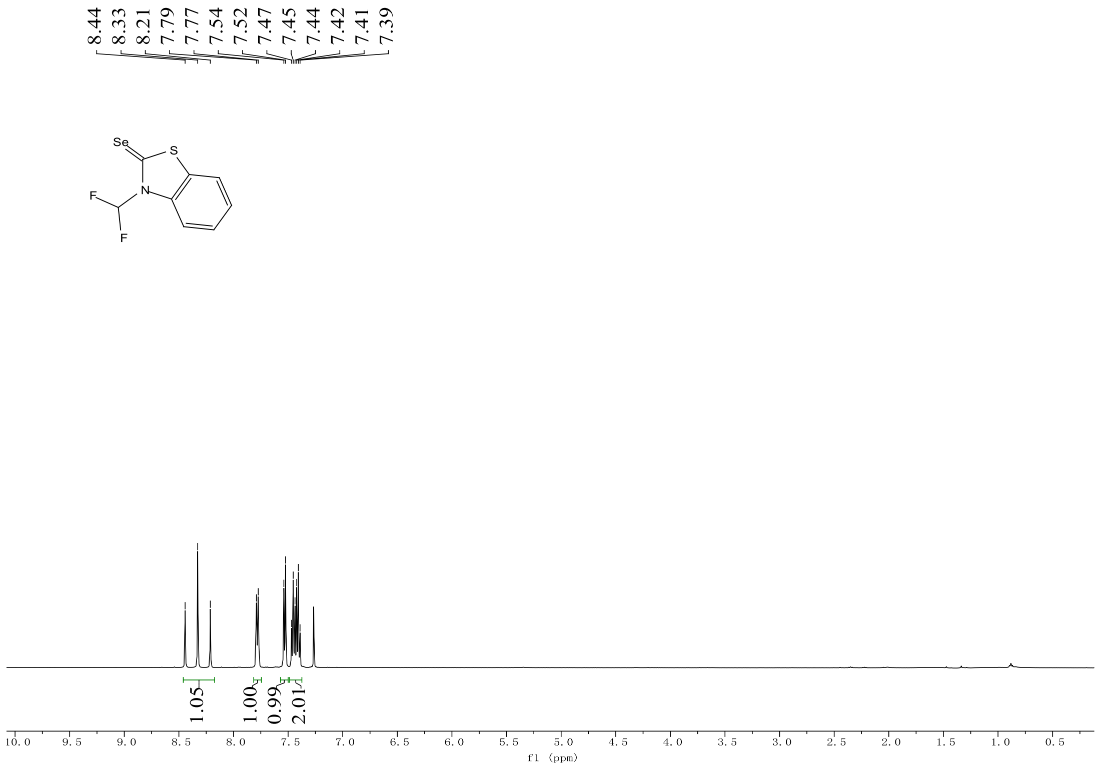

The ^{1}H NMR spectrum of compound **3a**

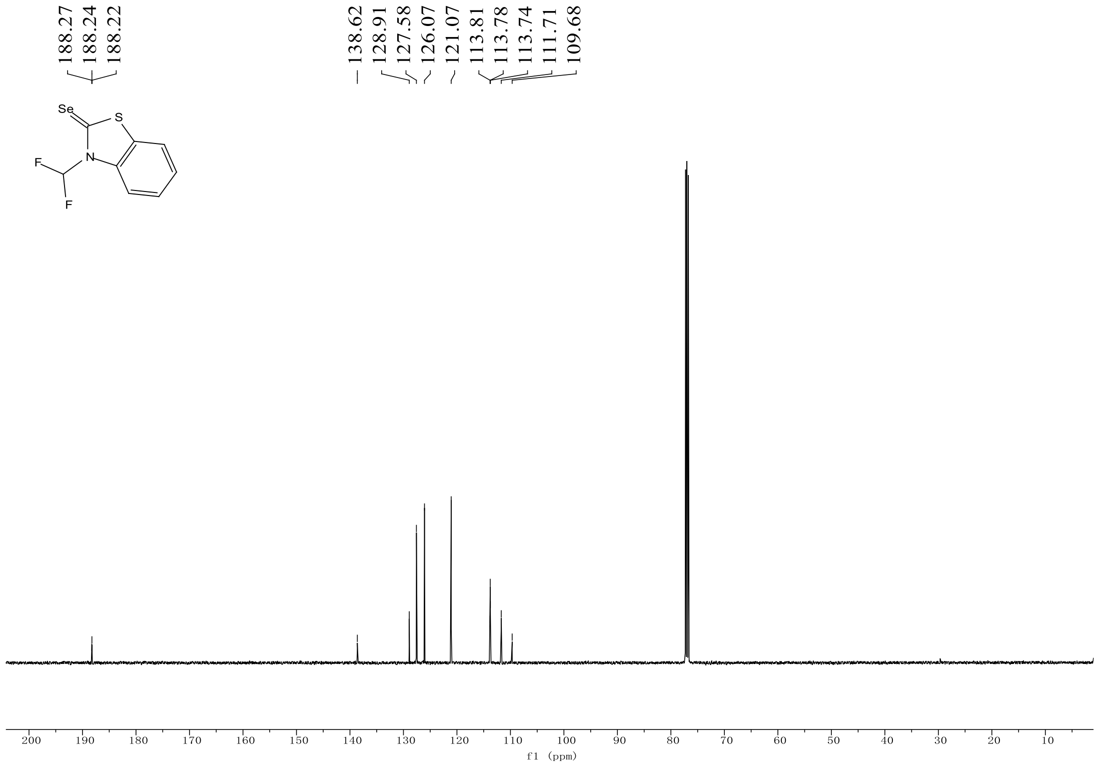

The ^{13}C NMR spectrum of compound **3a**

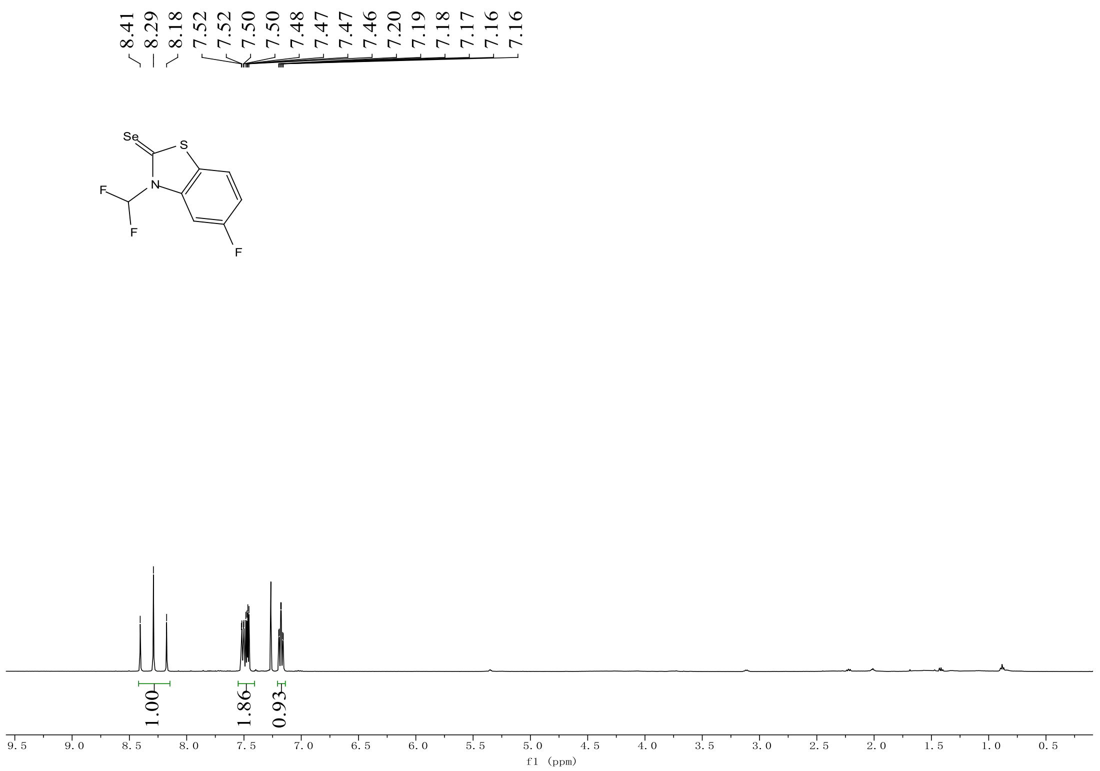

The ^{1}H NMR spectrum of compound **3b**

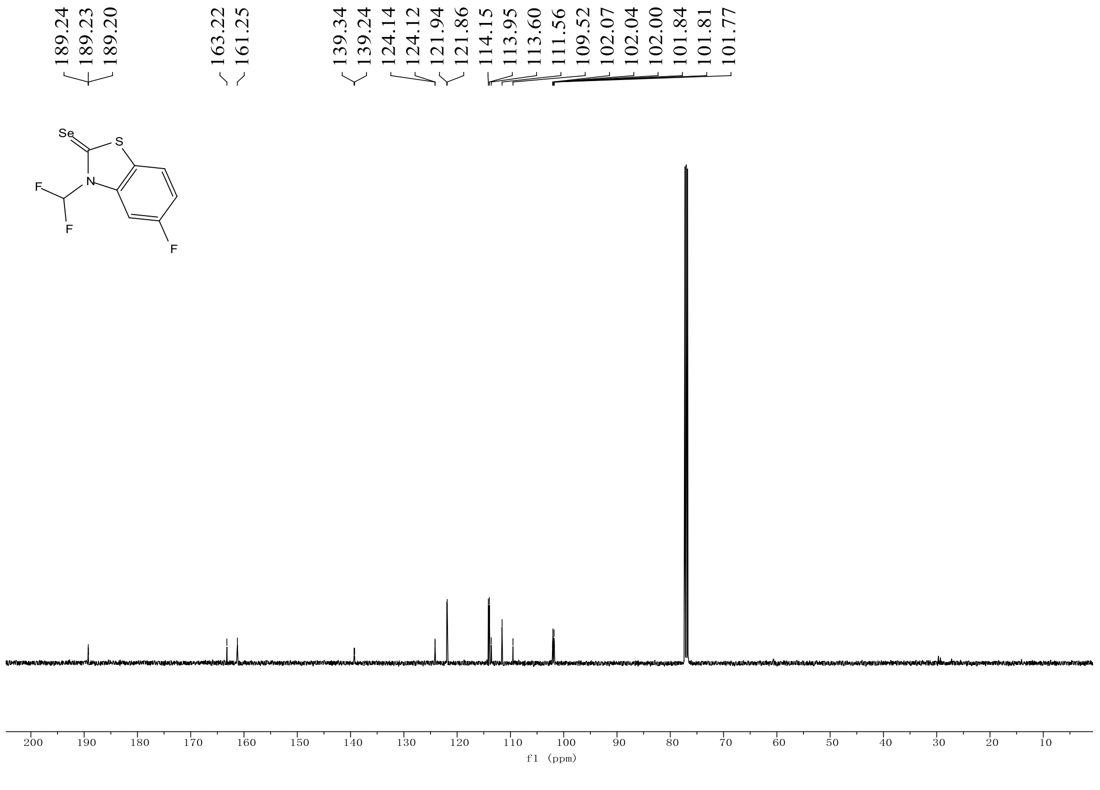

The ^{13}C NMR spectrum of compound **3b**

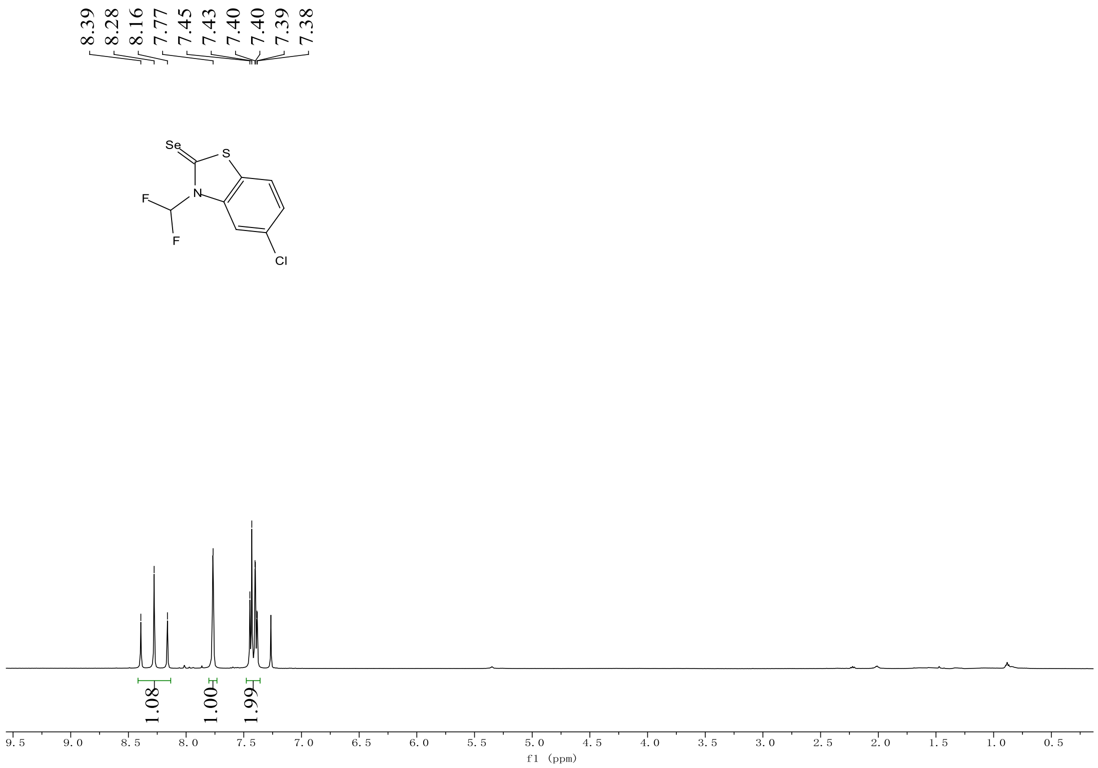

The ^{1}H NMR spectrum of compound **3c**

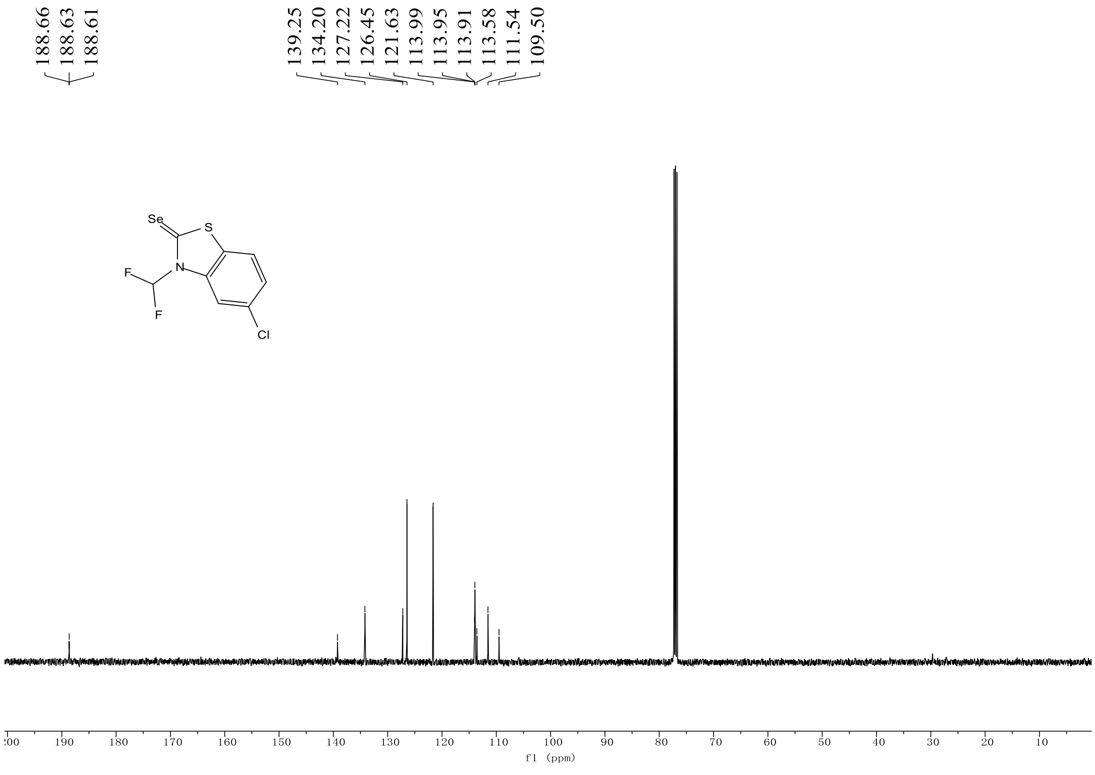

The ^{13}C NMR spectrum of compound **3c**

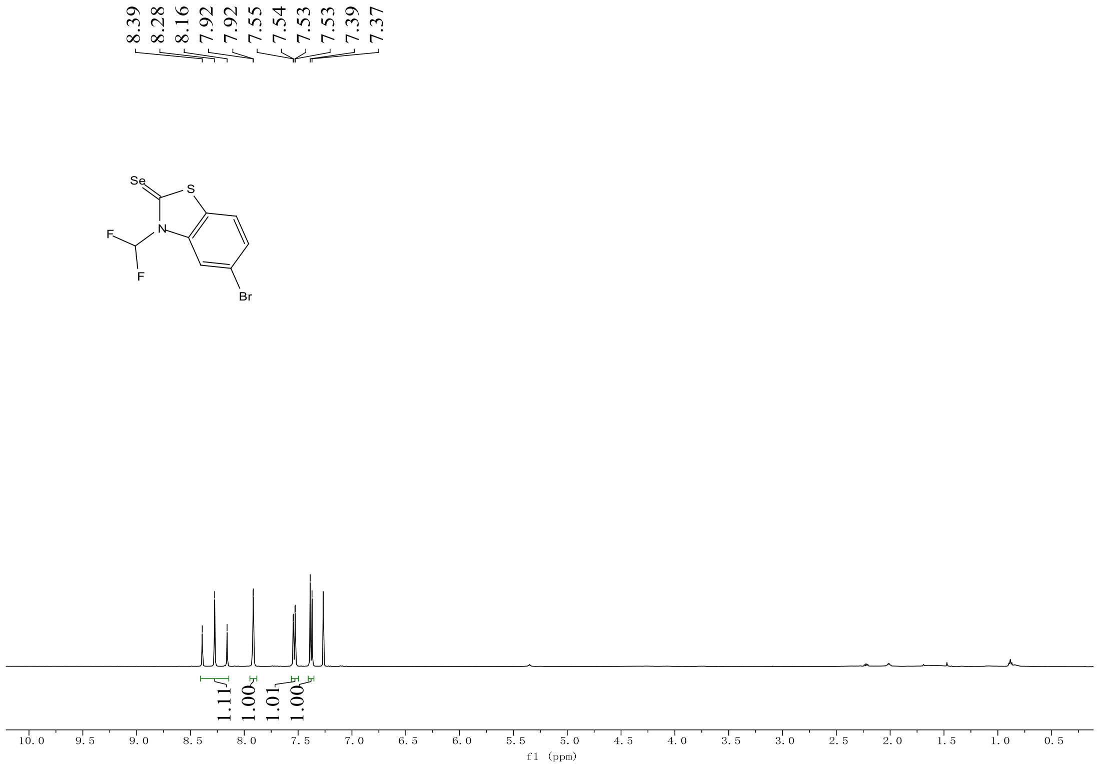

The ^{1}H NMR spectrum of compound **3d**

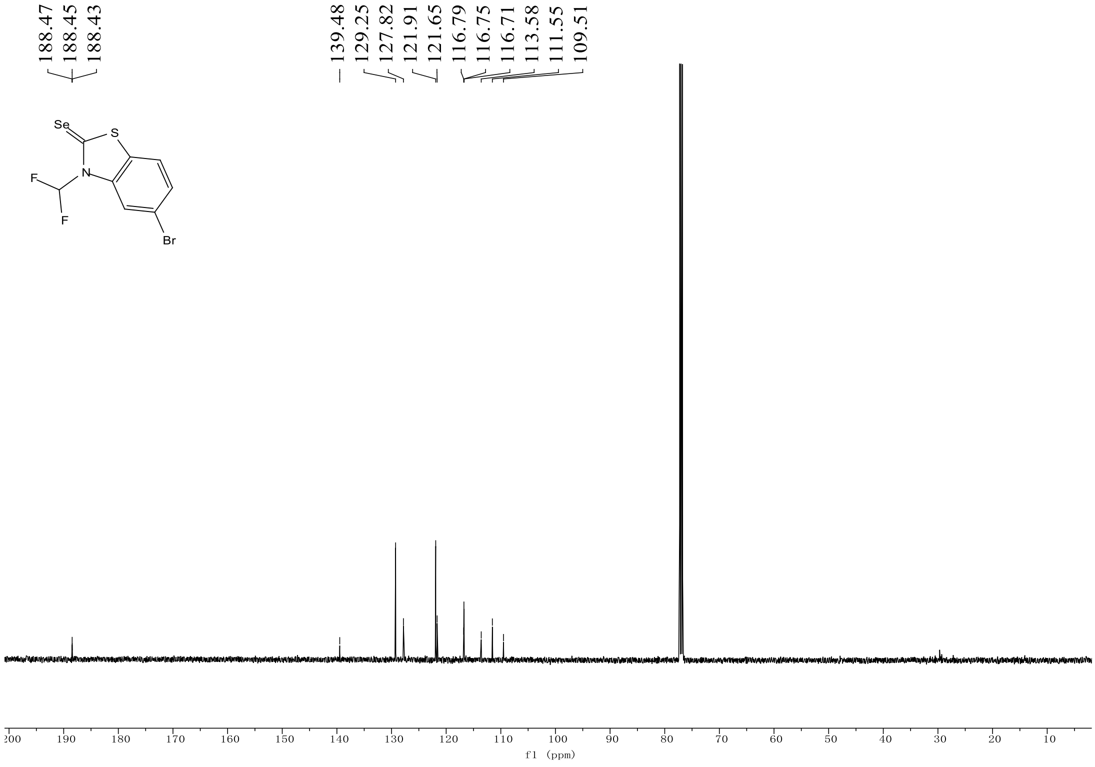

The ^{13}C NMR spectrum of compound **3d**

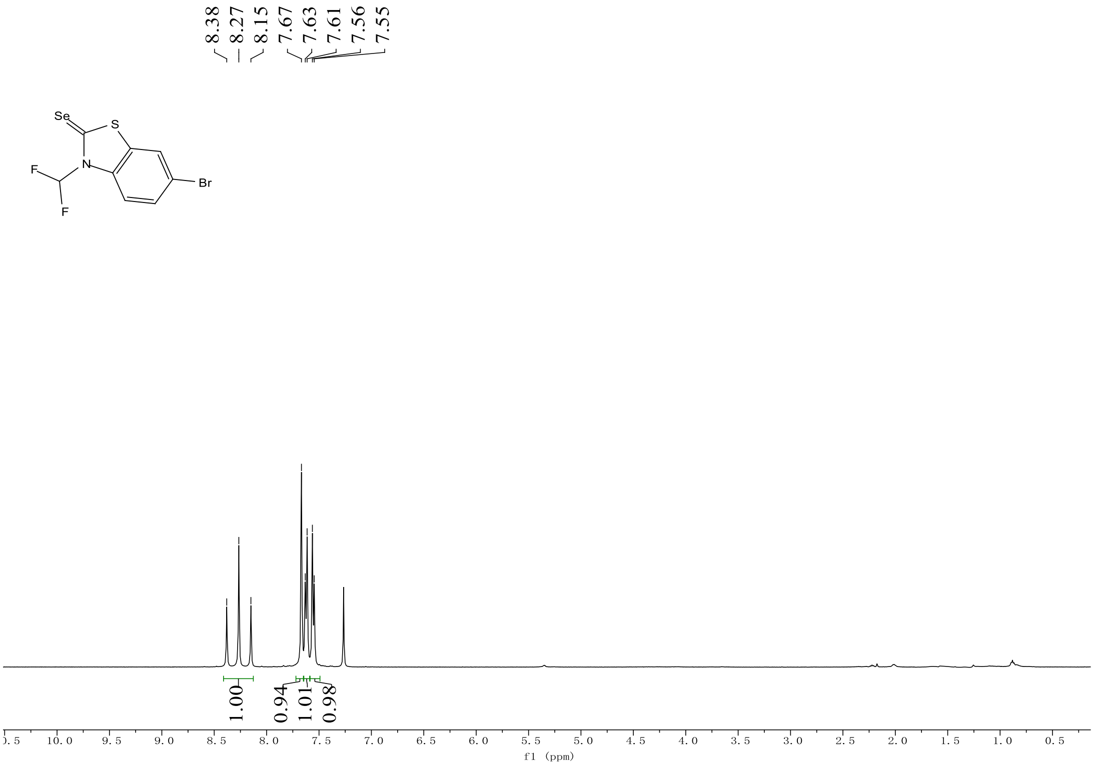

The ^{1}H NMR spectrum of compound **3e**

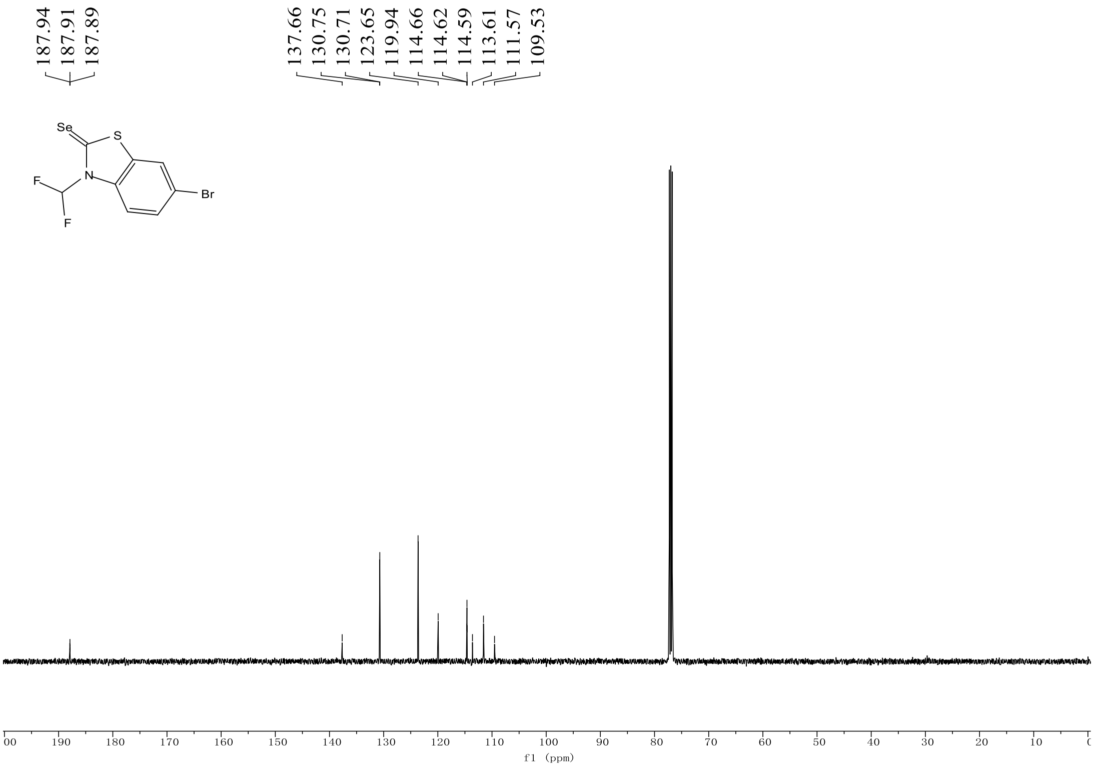

The ^{13}C NMR spectrum of compound **3e**

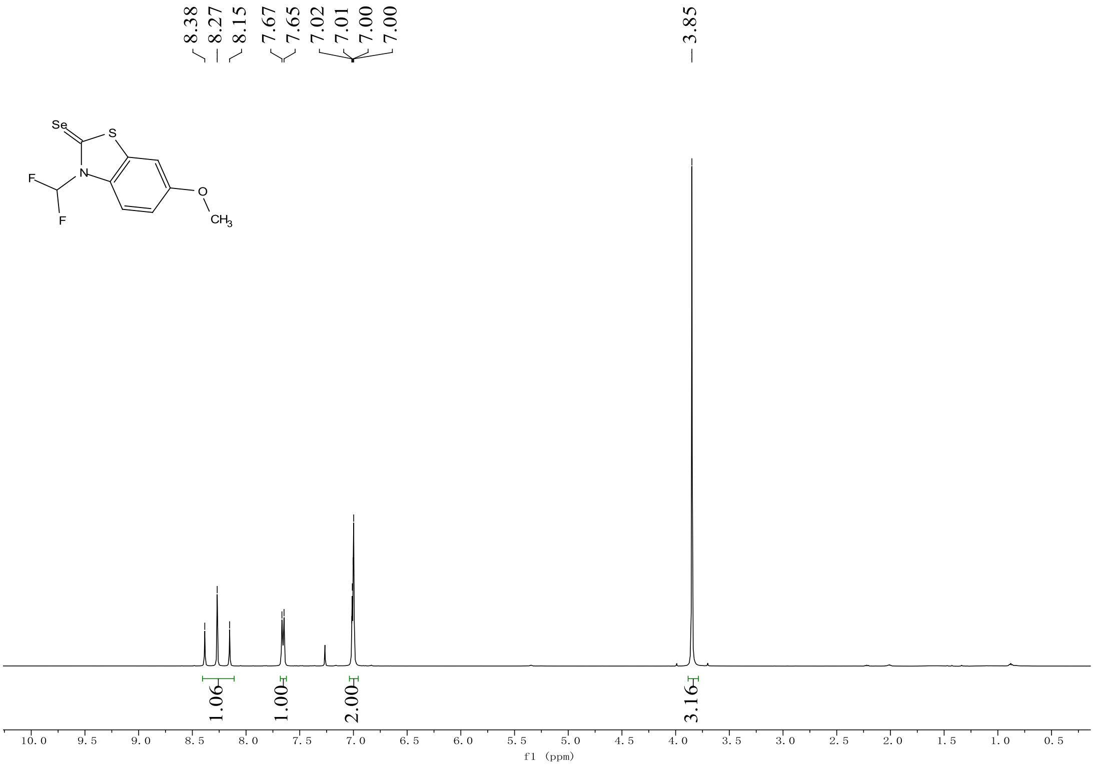

The ^{1}H NMR spectrum of compound **3f**

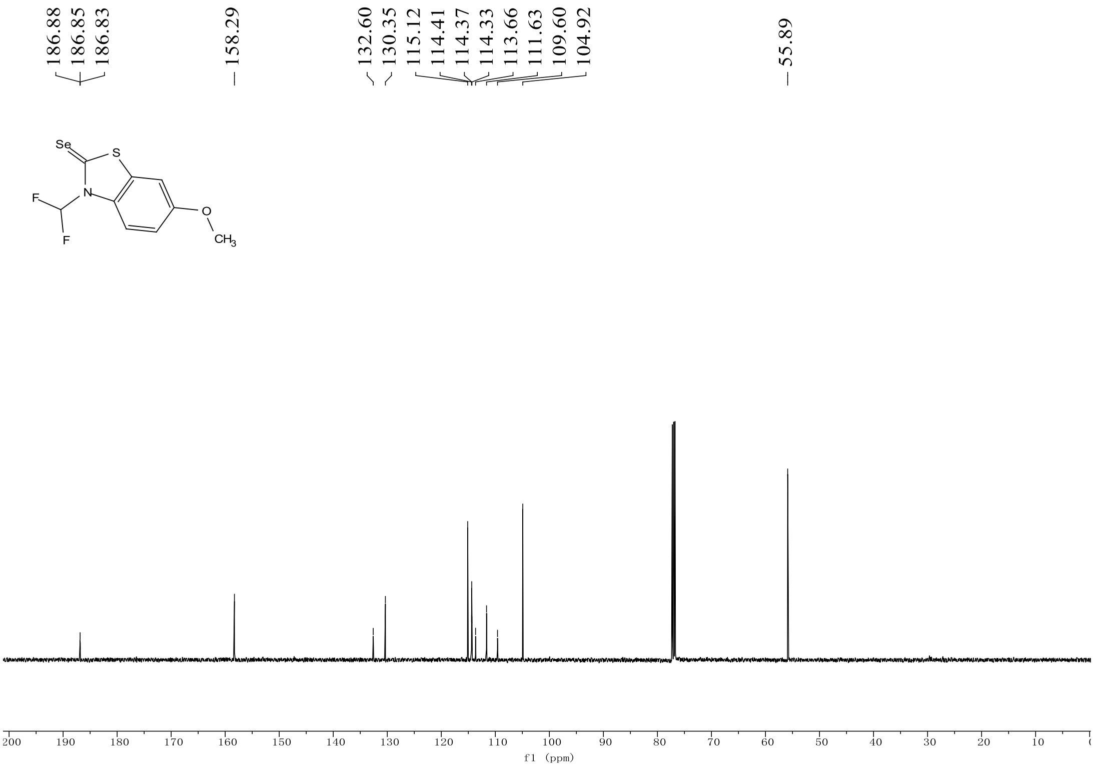

The ^{13}C NMR spectrum of compound **3f**

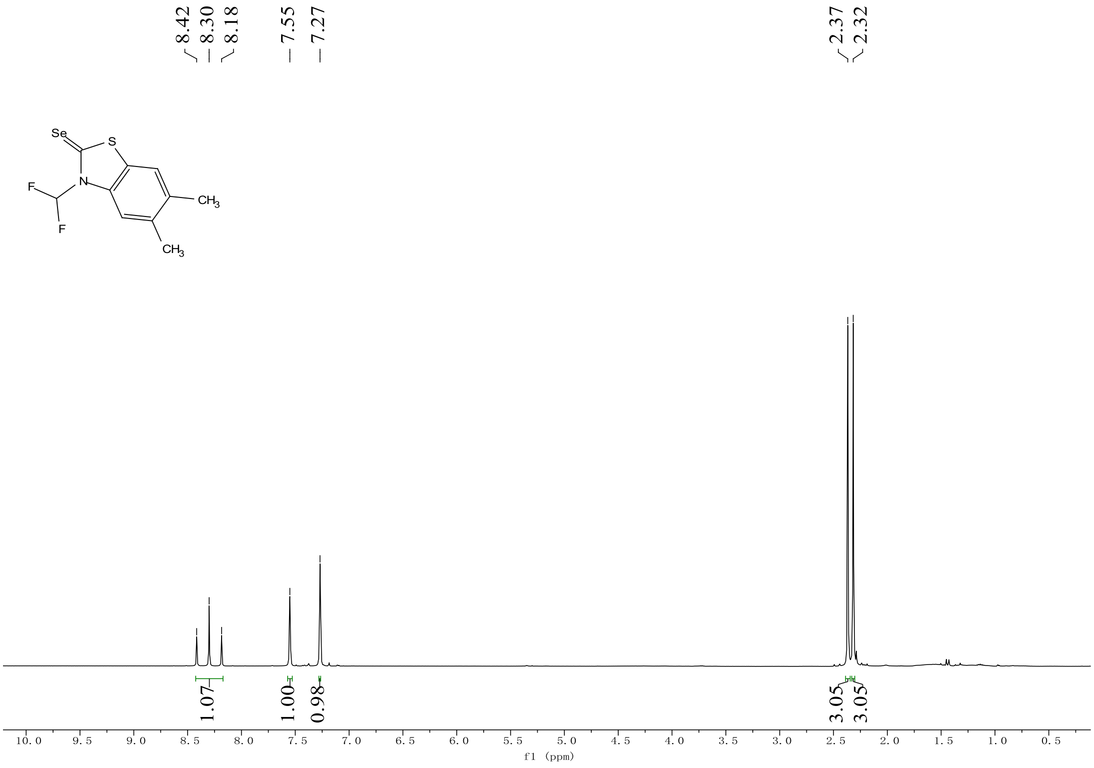

The ^{1}H NMR spectrum of compound **3g**

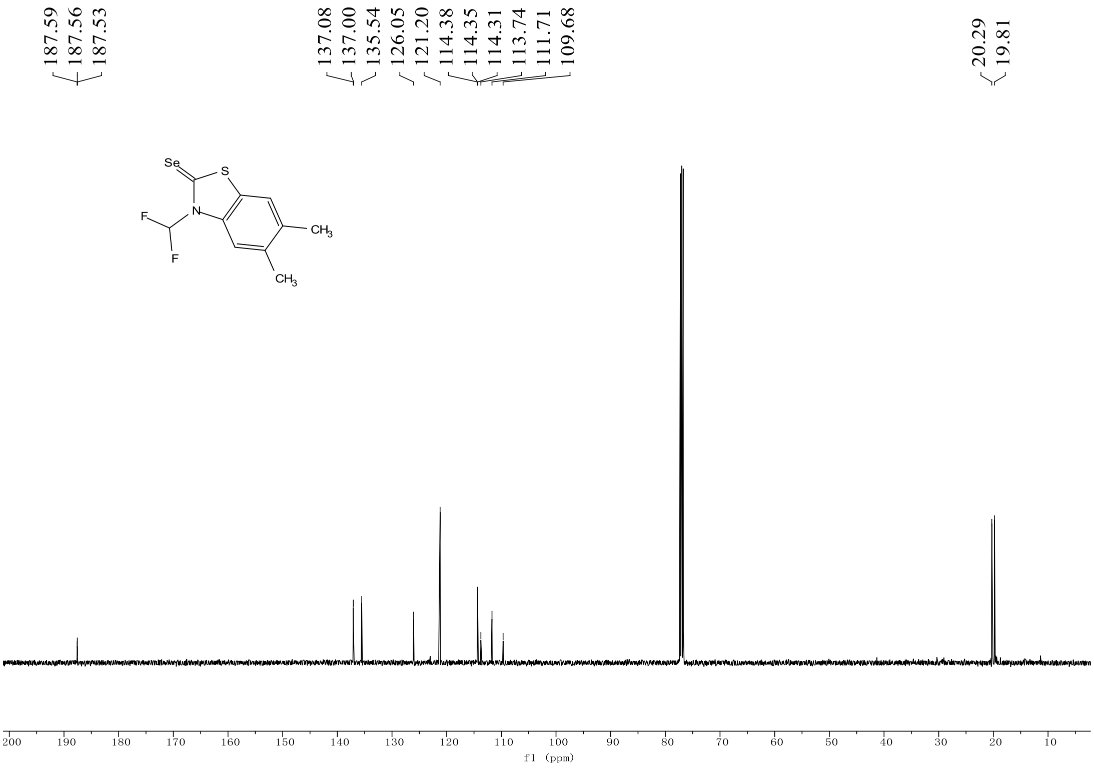

The ^{13}C NMR spectrum of compound **3g**

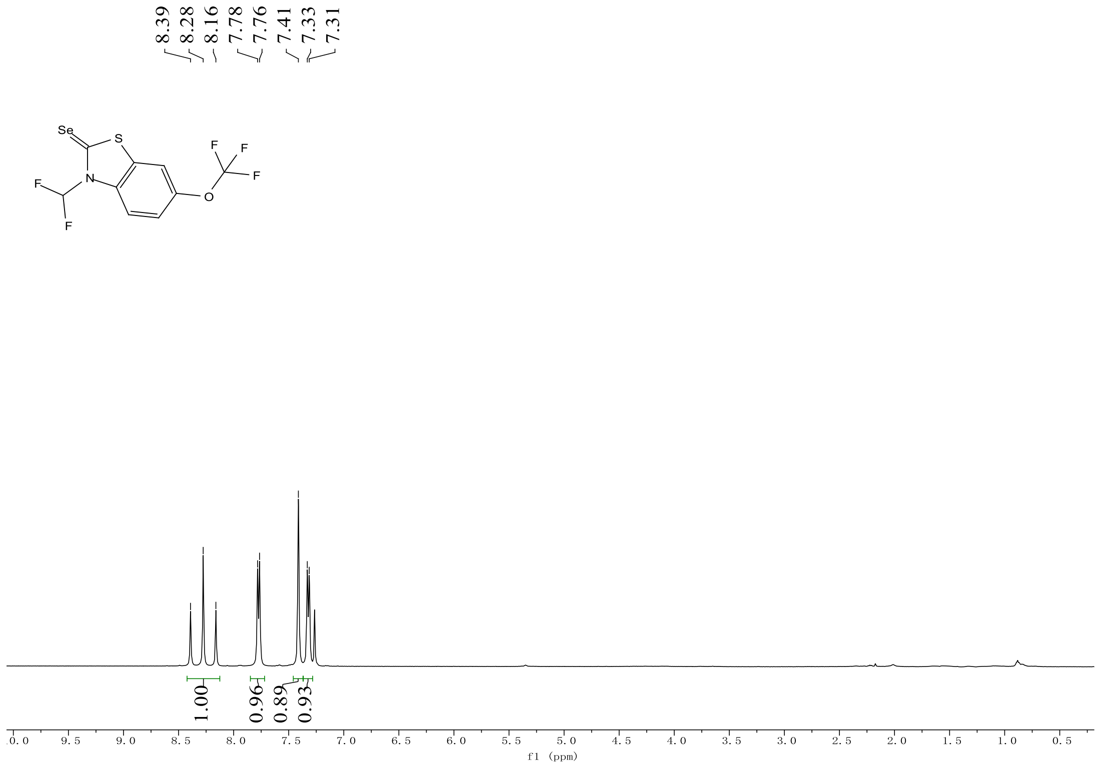

The ^{1}H NMR spectrum of compound **3h**

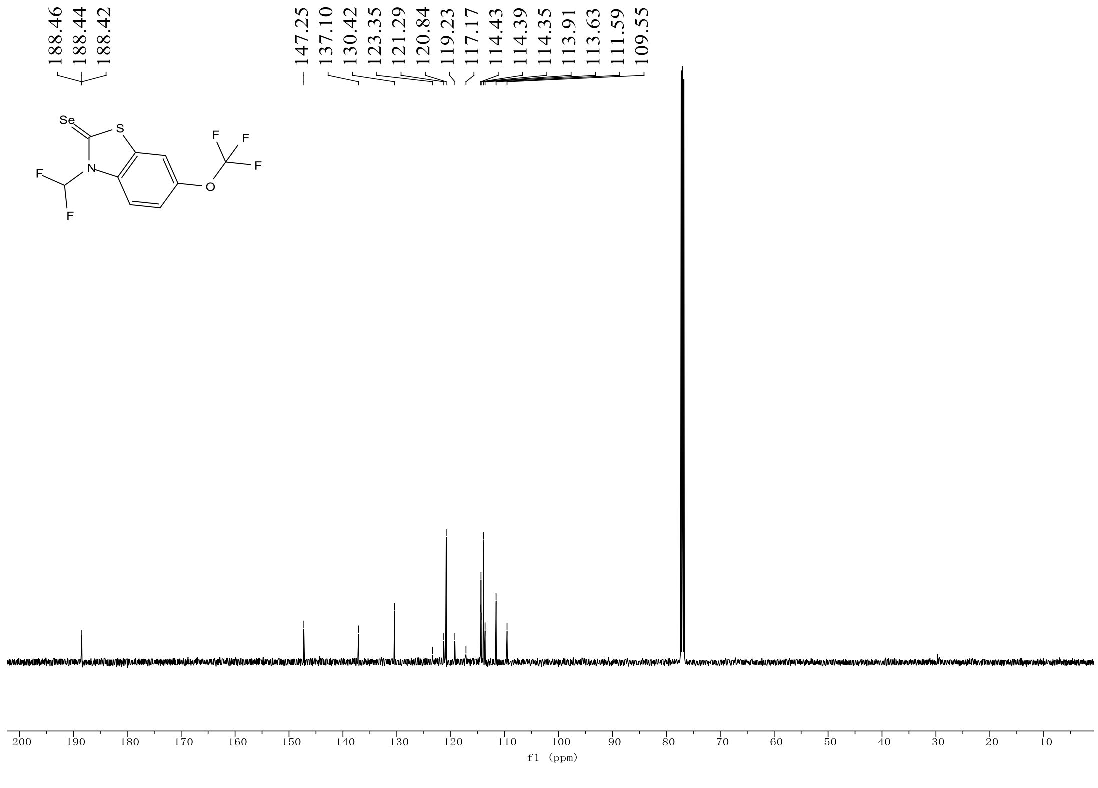

The ^{13}C NMR spectrum of compound **3h**

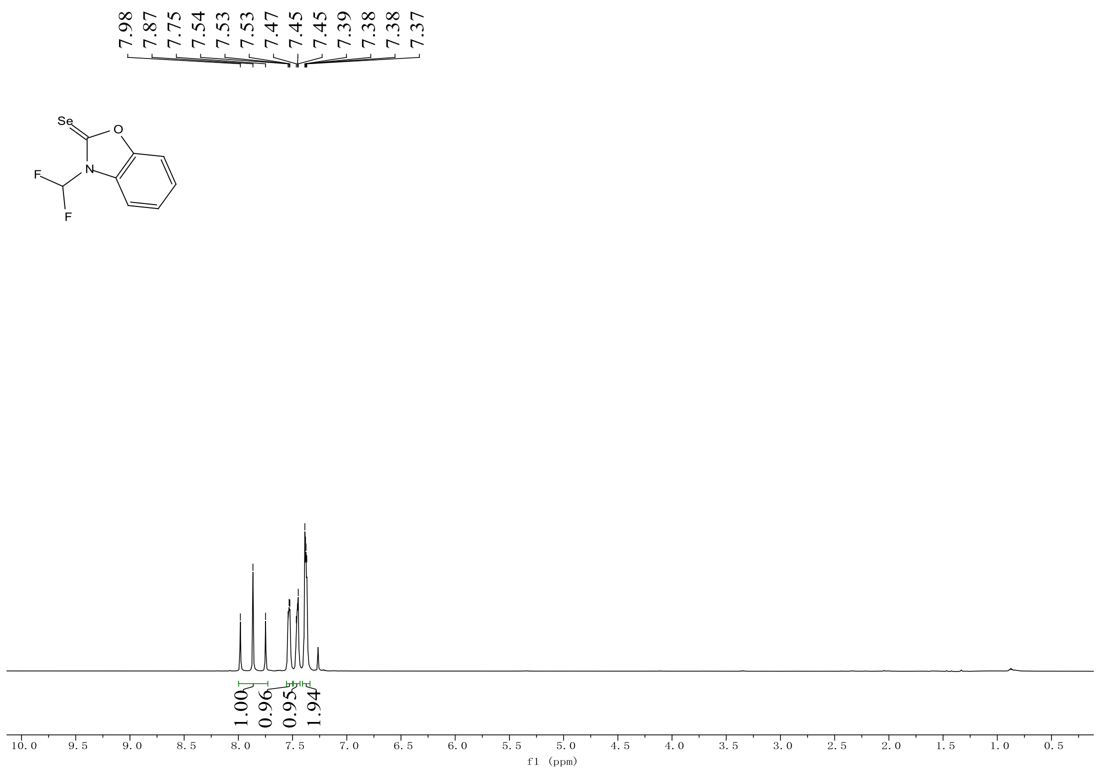

The ^{1}H NMR spectrum of compound **3i**

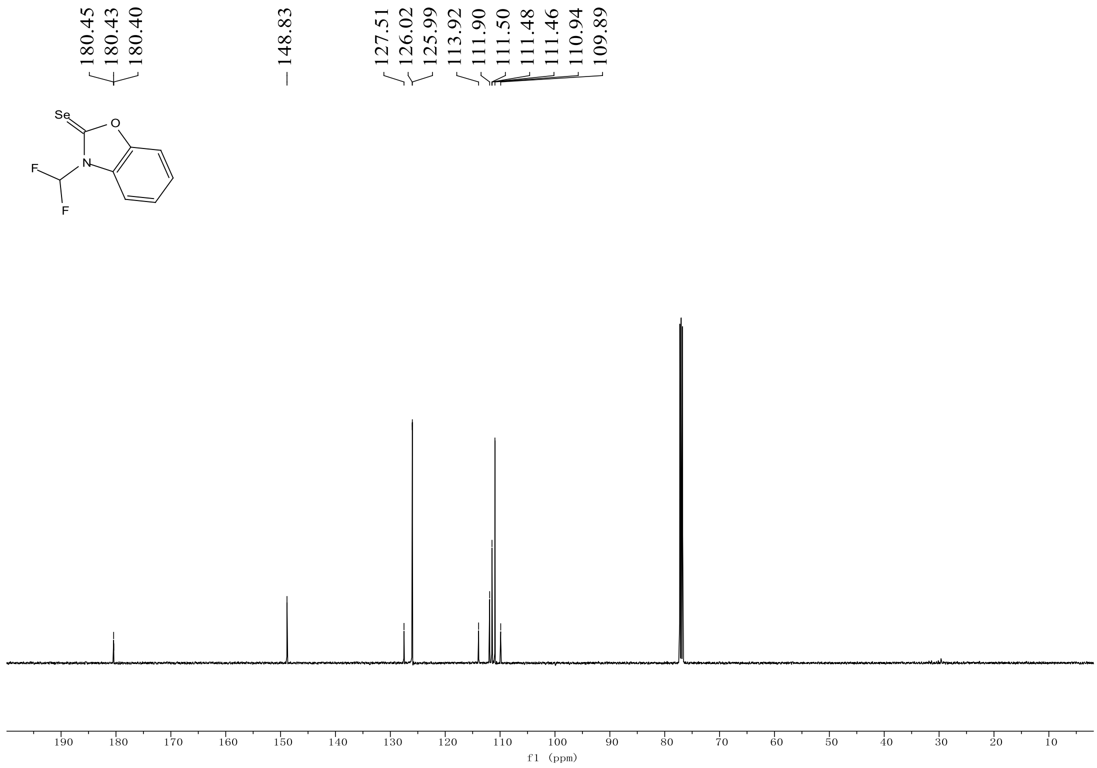

The ^{13}C NMR spectrum of compound **3i**

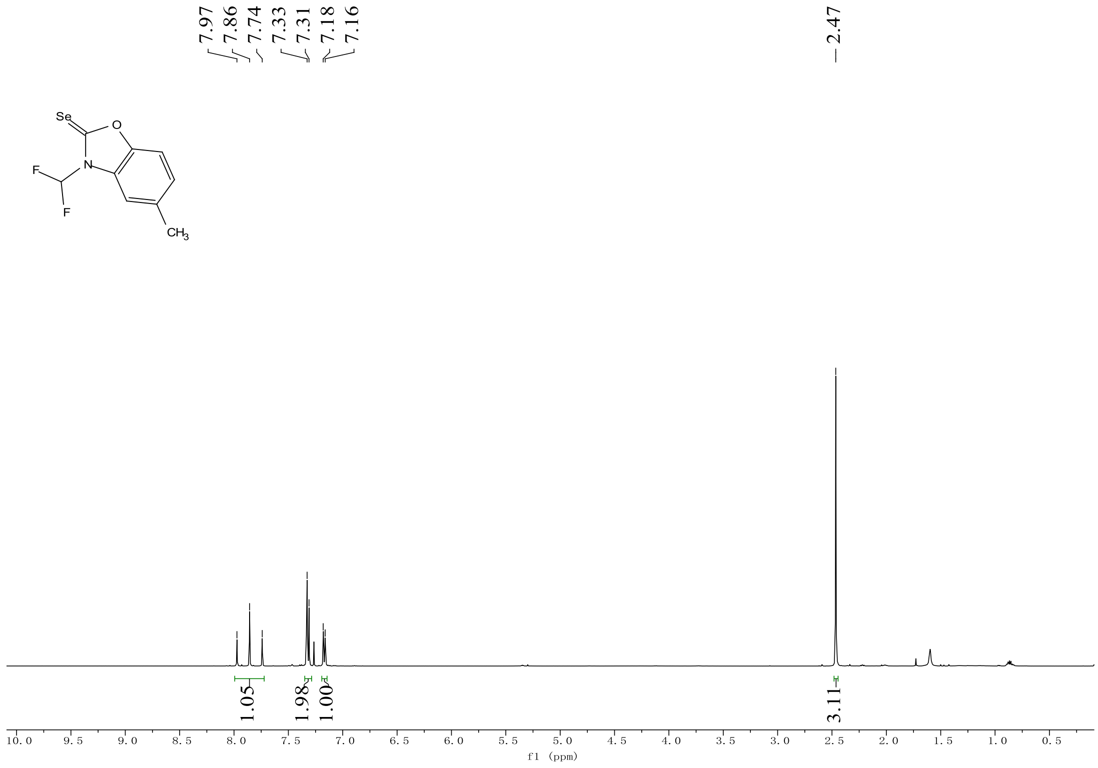

The ^{1}H NMR spectrum of compound **3j**

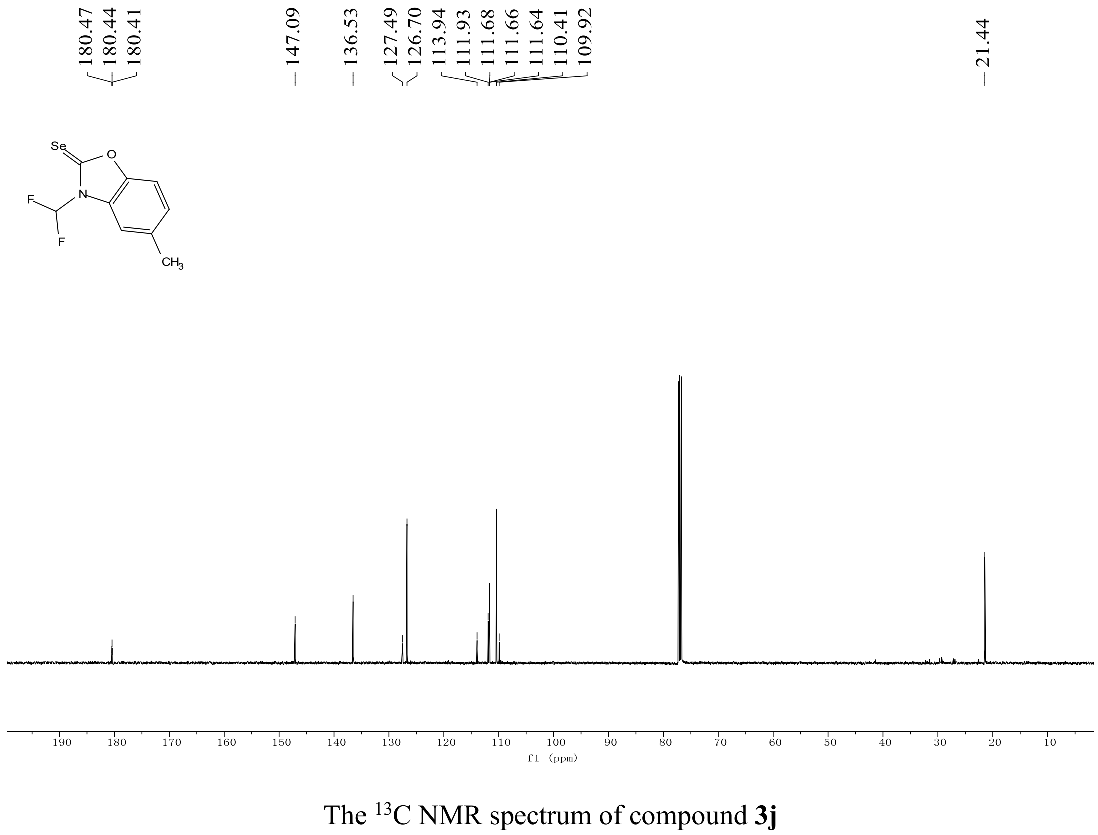

The ^{13}C NMR spectrum of compound **3j**

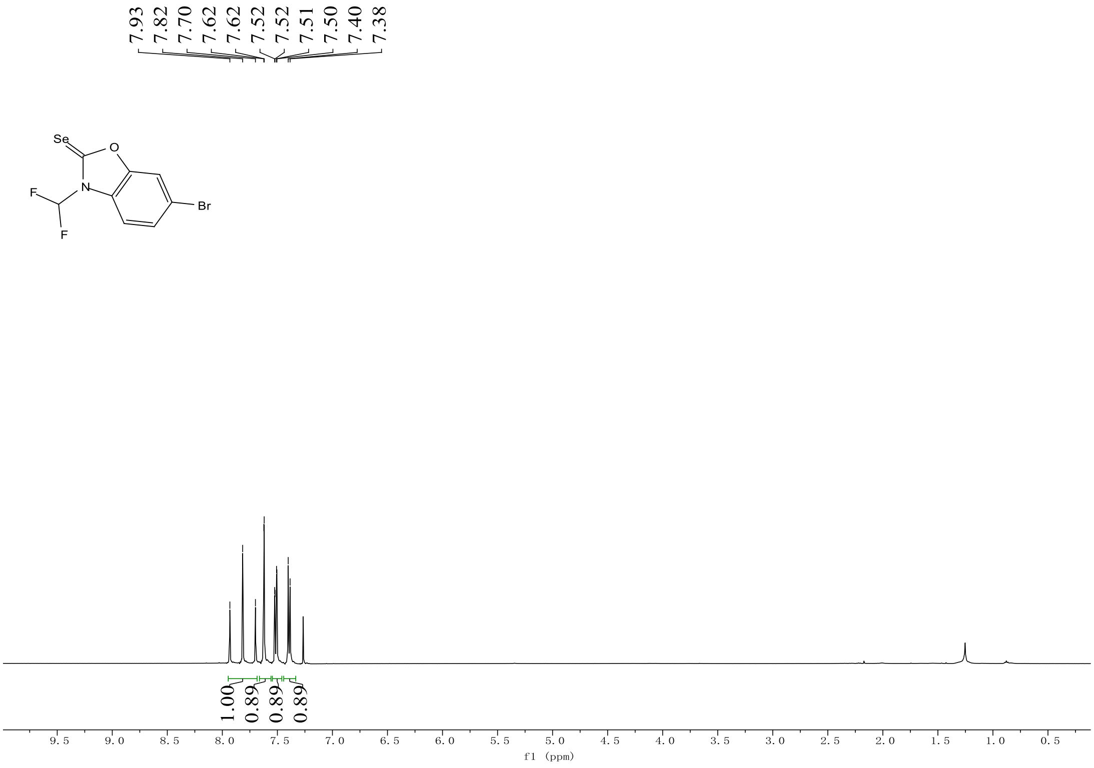

The ^{1}H NMR spectrum of compound **3k**

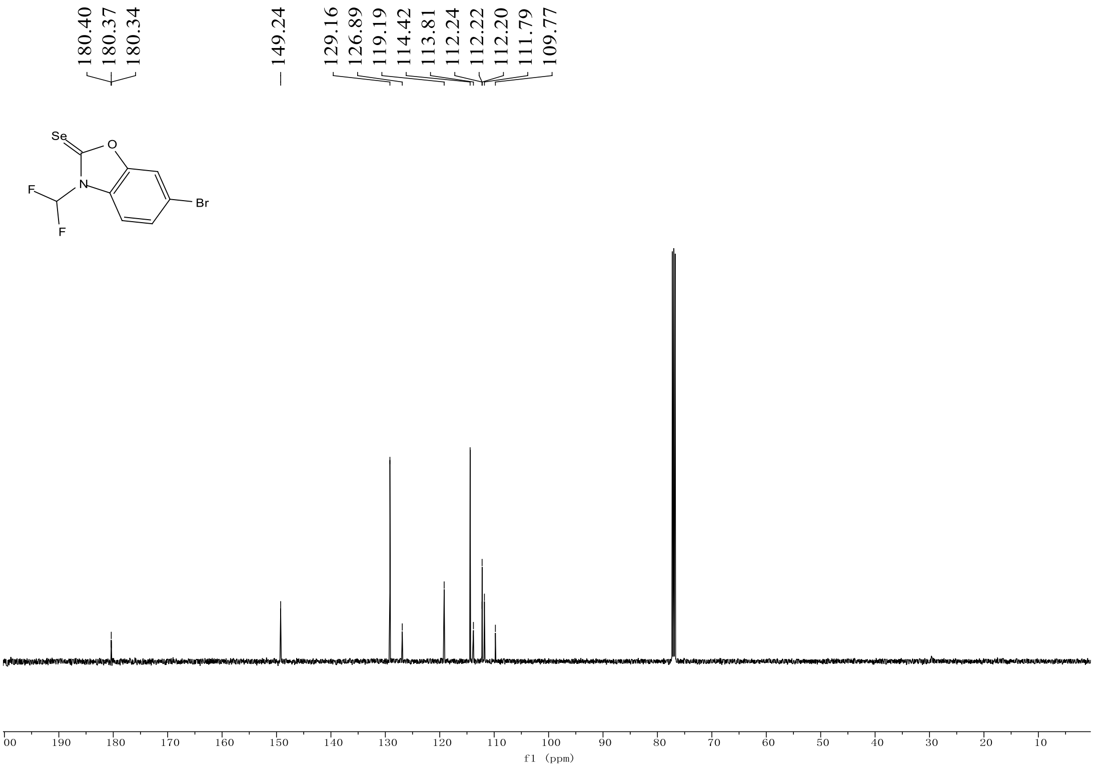

The ^{13}C NMR spectrum of compound **3k**

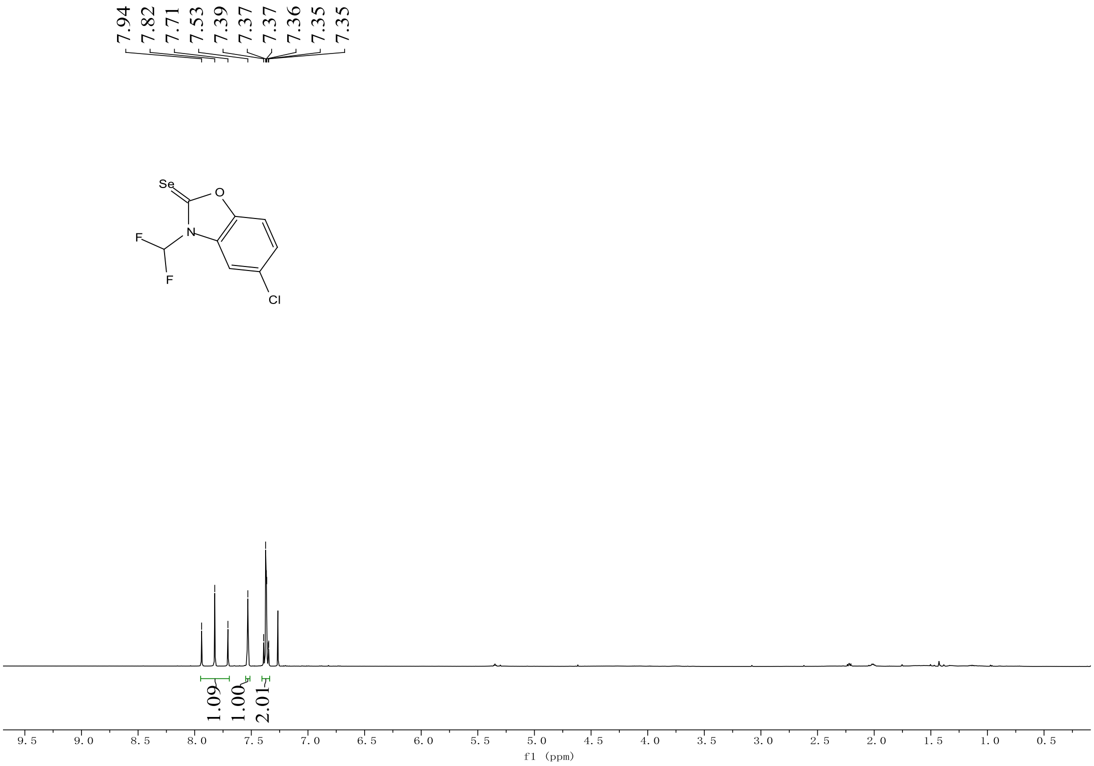

The ^{1}H NMR spectrum of compound **3l**

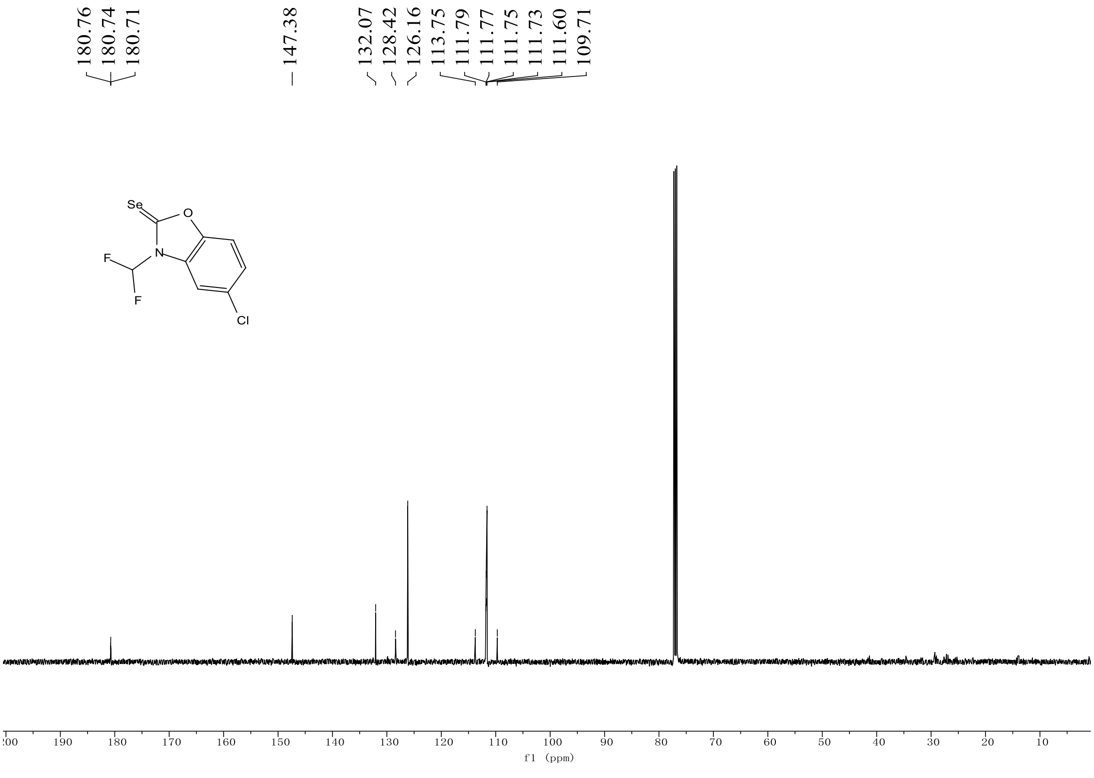

The ^{13}C NMR spectrum of compound **3l**

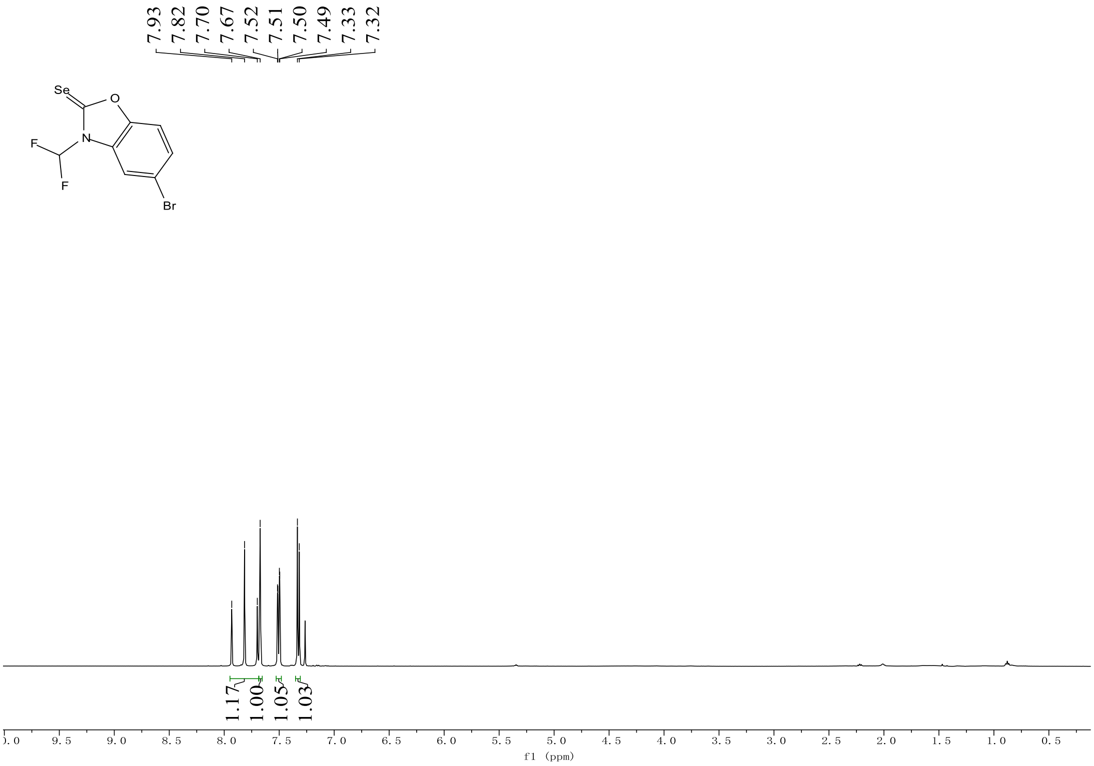

The ^{1}H NMR spectrum of compound **3m**

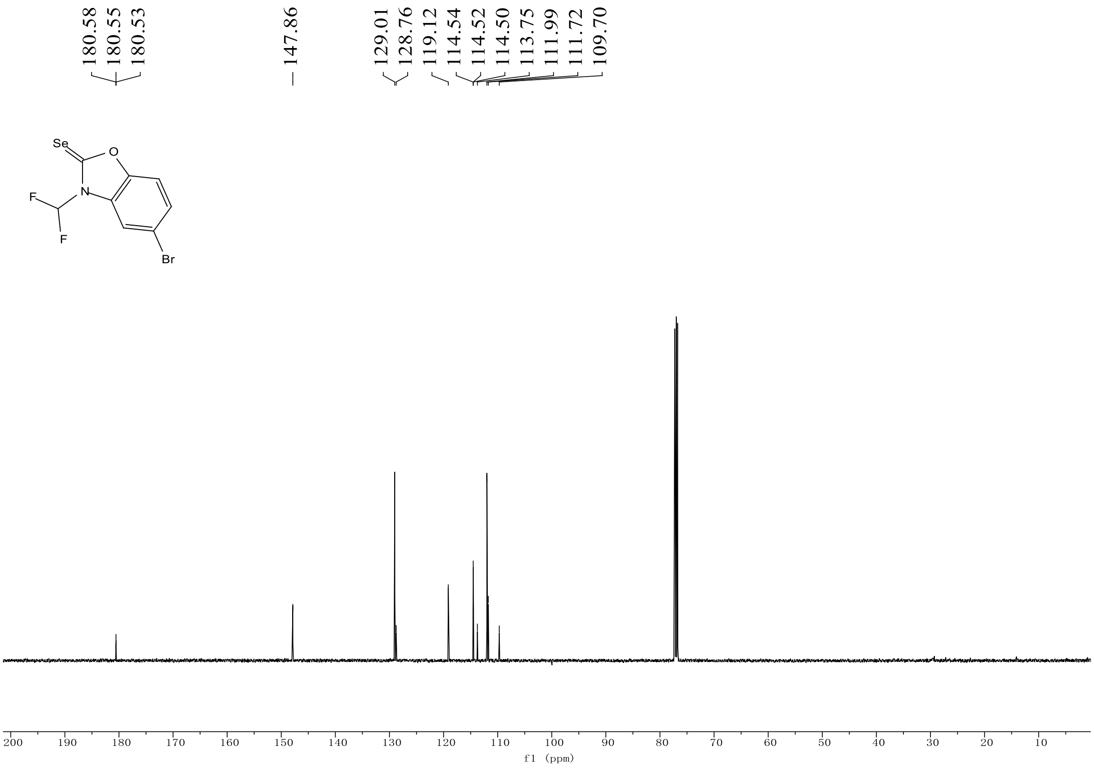

The ^{13}C NMR spectrum of compound **3m**

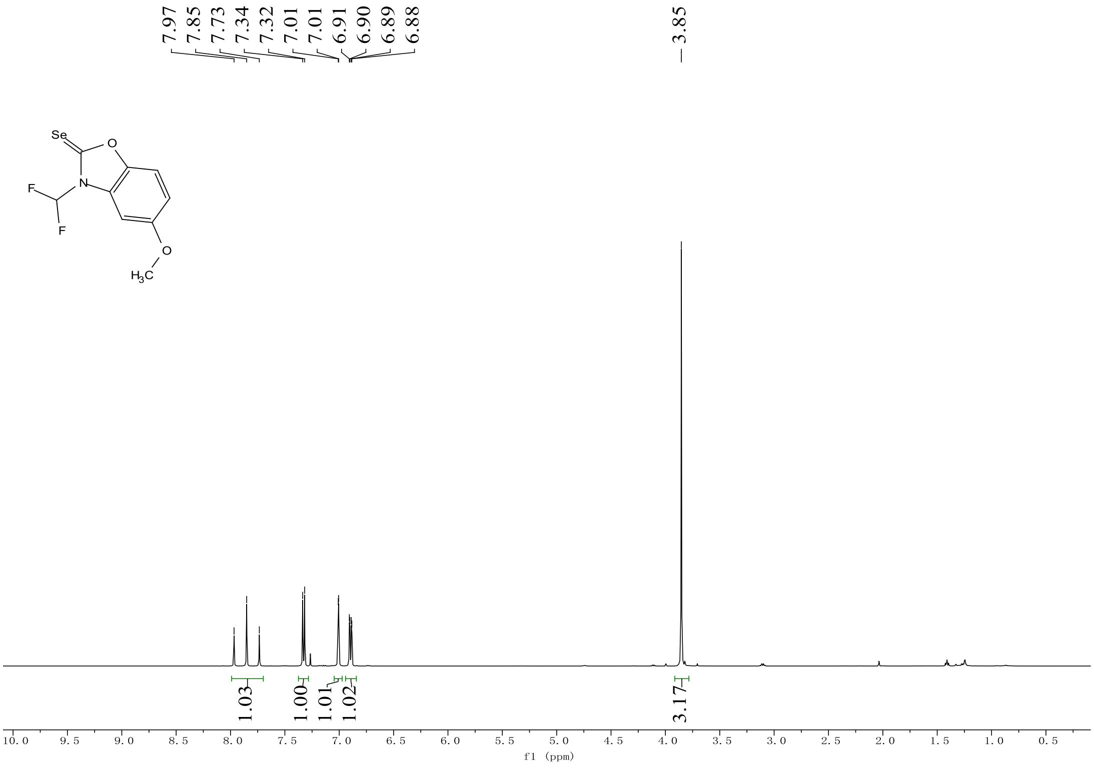

The ^{1}H NMR spectrum of compound **3n**

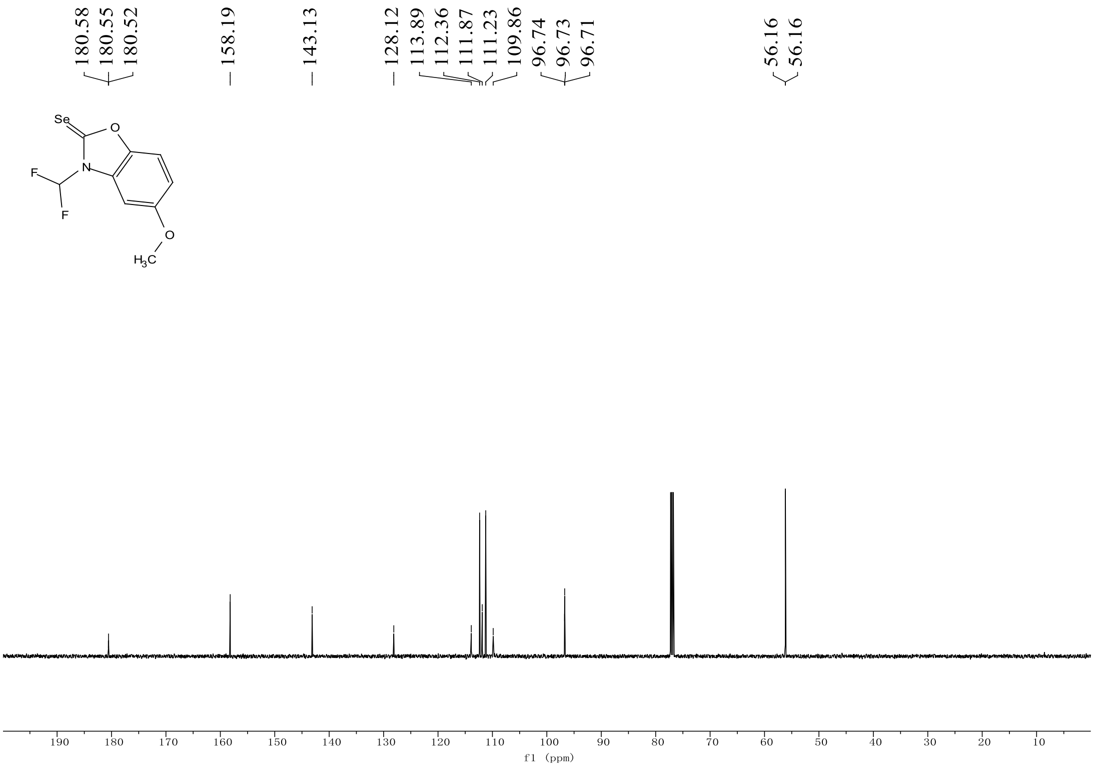

The ^{13}C NMR spectrum of compound **3n**

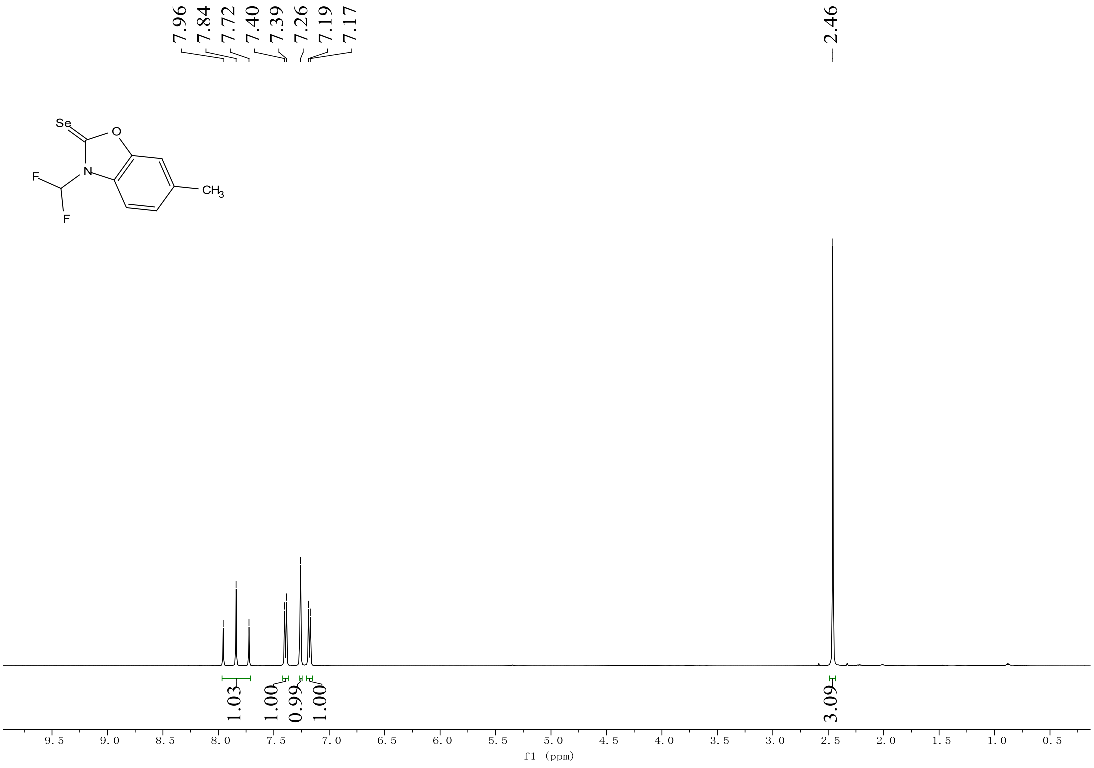

The ^{1}H NMR spectrum of compound **3o**

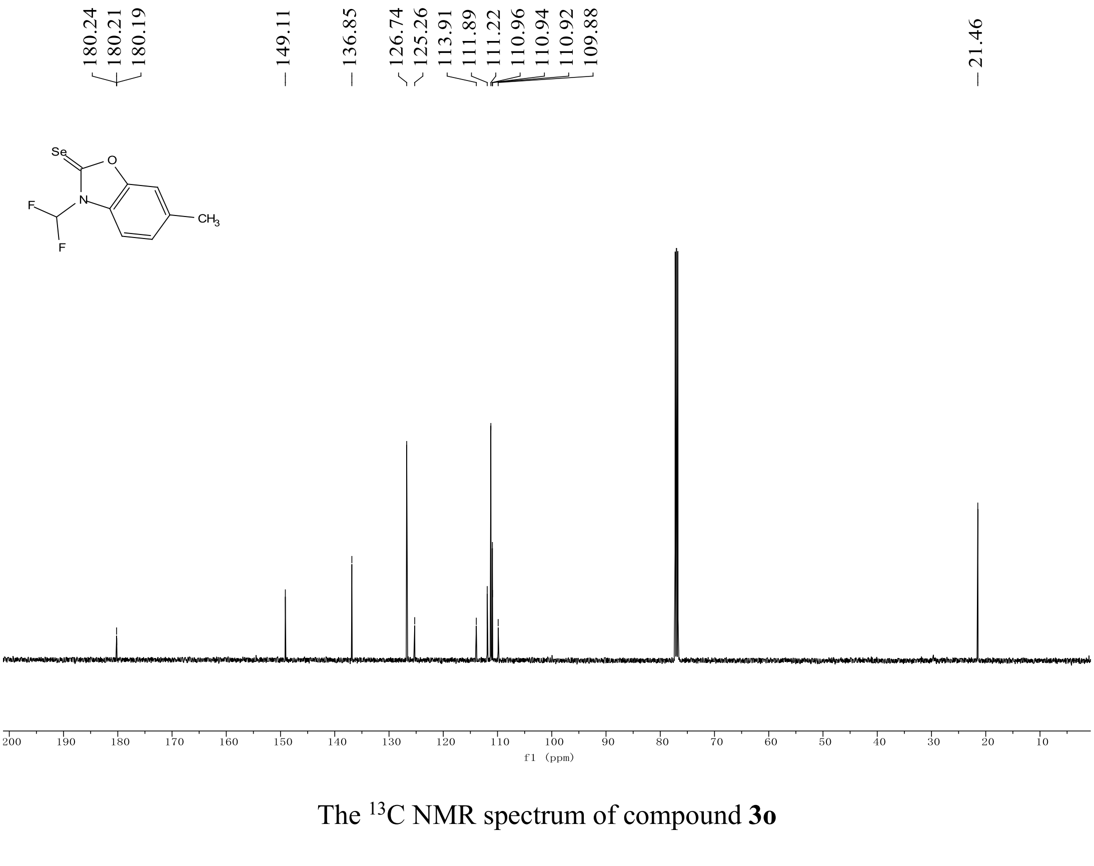

The ^{13}C NMR spectrum of compound **3o**

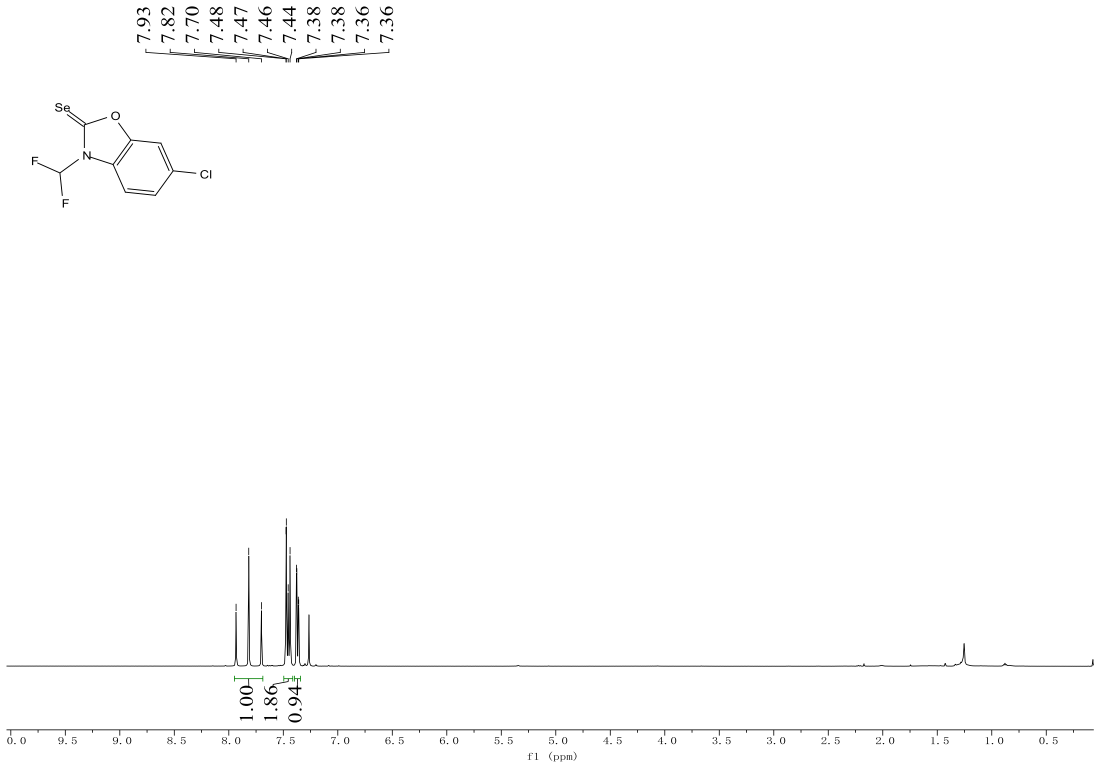

The ^{1}H NMR spectrum of compound **3p**

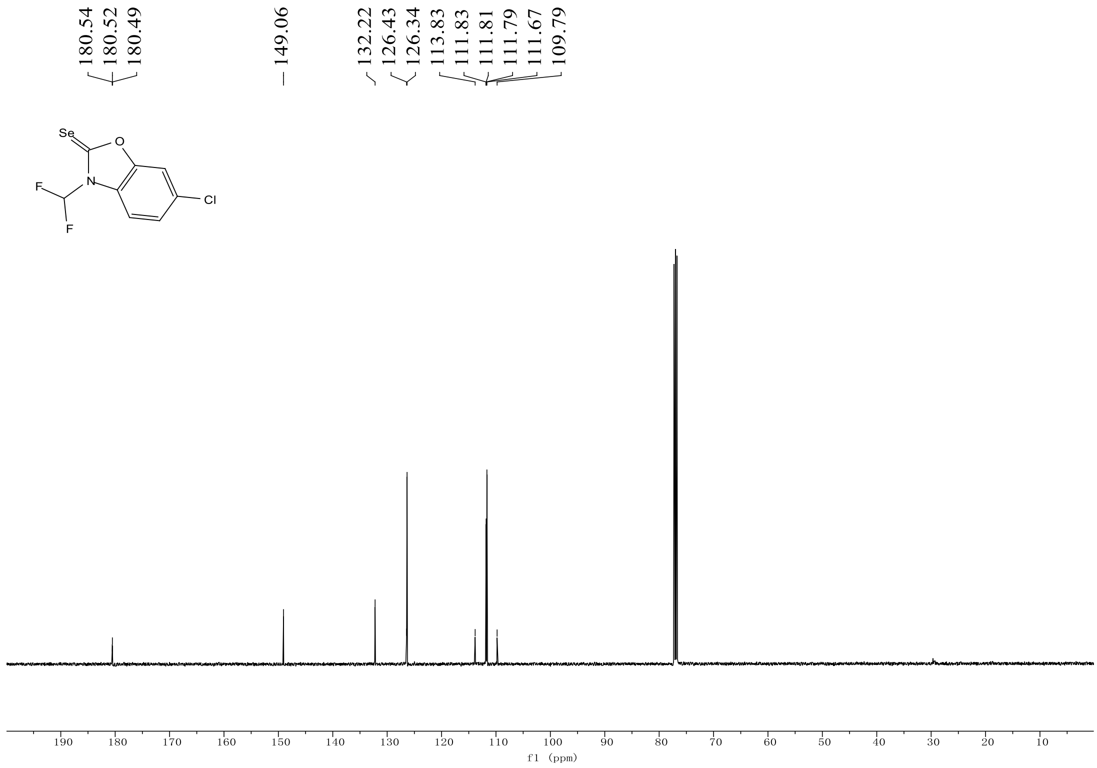

The ^{13}C NMR spectrum of compound **3p**

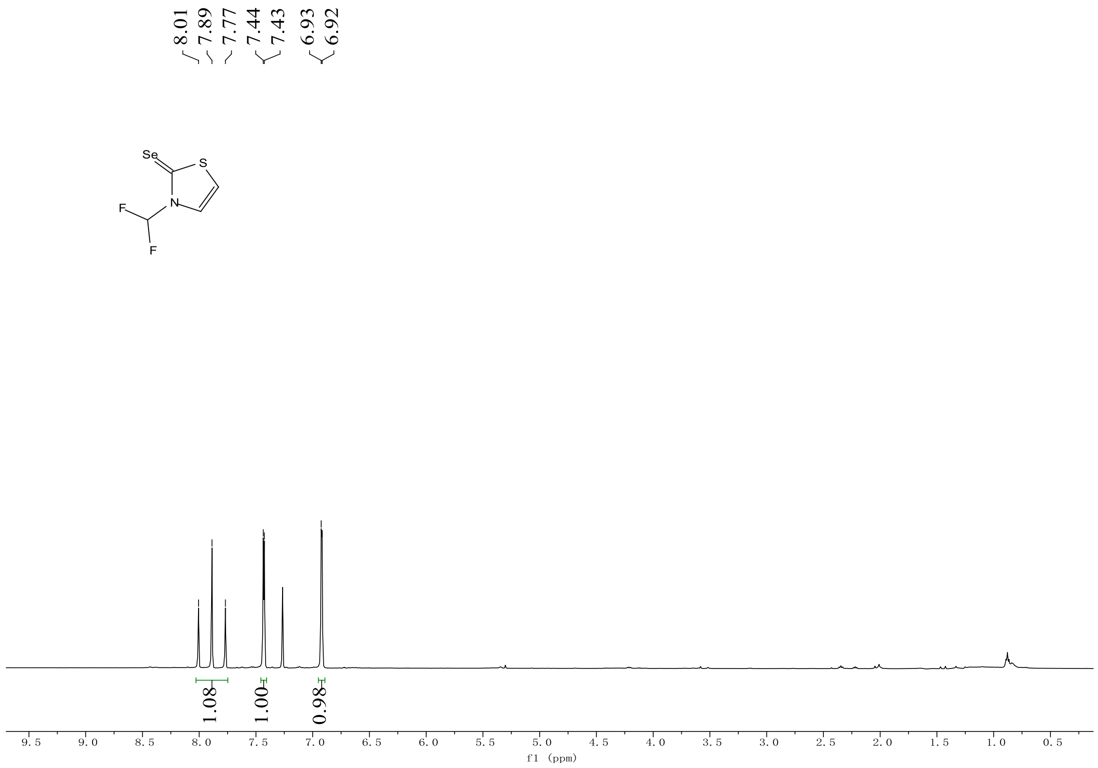

The ^{1}H NMR spectrum of compound 4

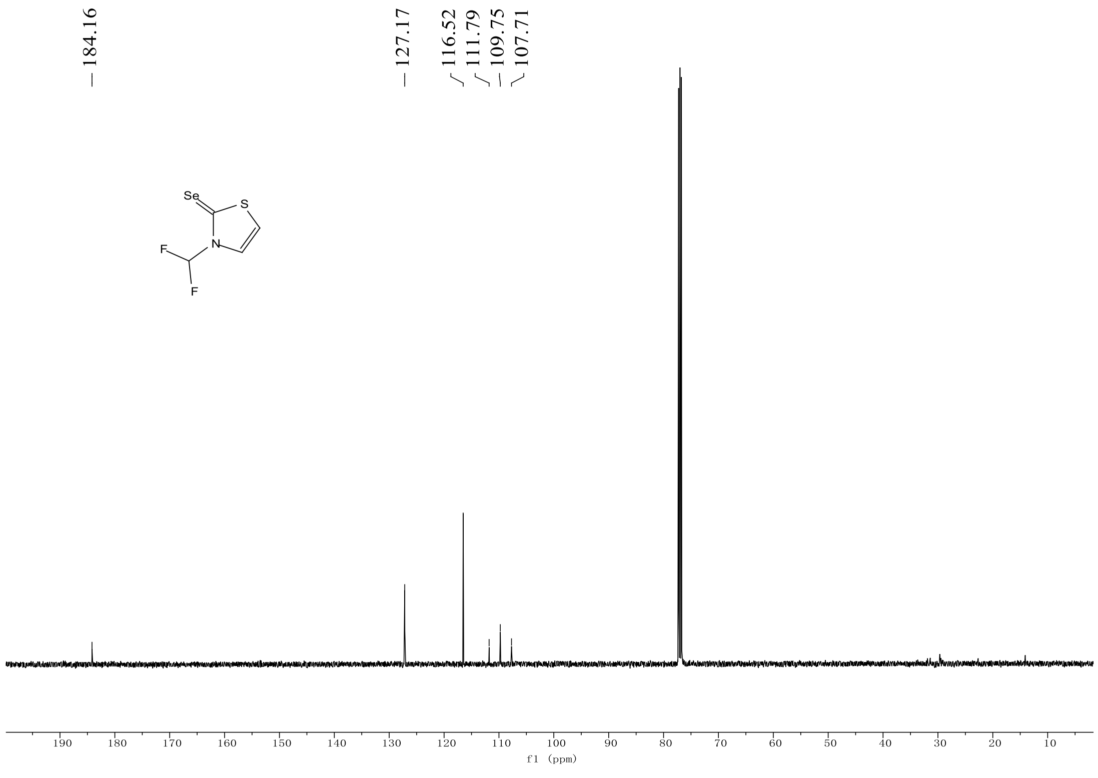

The ^{13}C NMR spectrum of compound 4

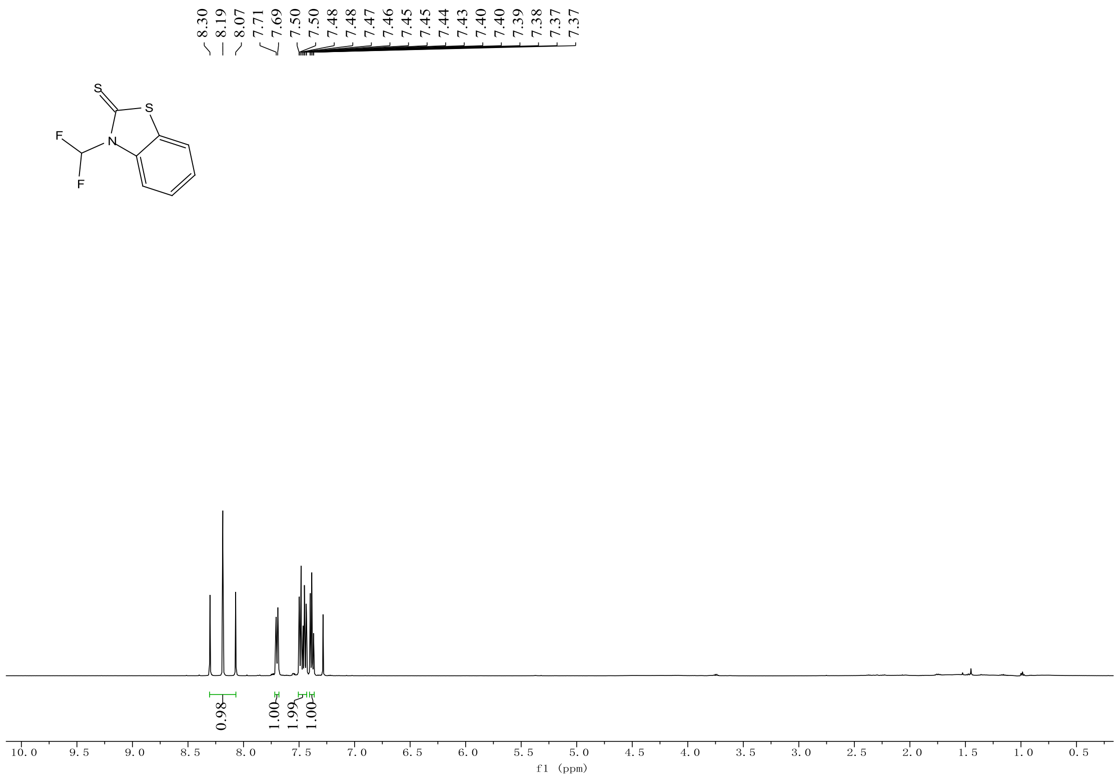

The ^{1}H NMR spectrum of compound **6**

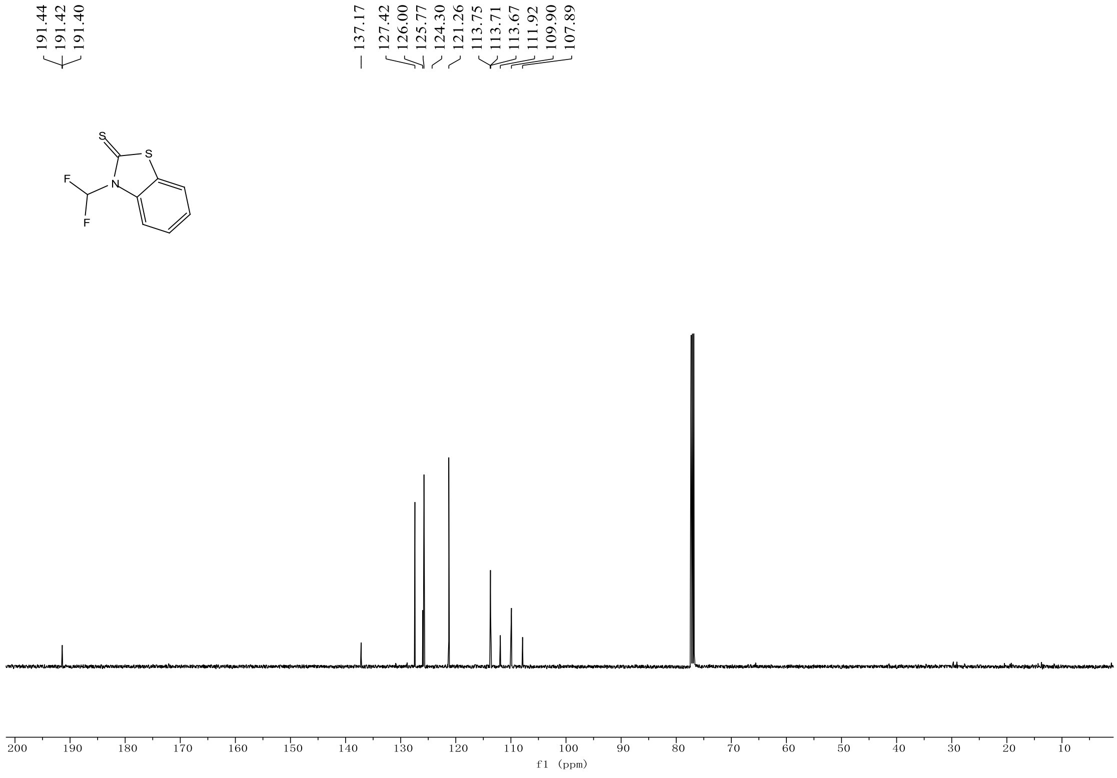

The ^{13}C NMR spectrum of compound **6**
